# PC-mer: An Ultra-fast memory-efficient tool for metagenomics profiling and classification

**DOI:** 10.1371/journal.pone.0307279

**Published:** 2024-08-01

**Authors:** Saeedeh Akbari Rokn Abadi, Amirhossein Mohammadi, Somayyeh Koohi

**Affiliations:** Department of Computer Engineering, Sharif University of Technology, Tehran, Iran; University of Enna Kore: Universita degli Studi di Enna ’Kore’, ITALY

## Abstract

Features extraction methods, such as k-mer-based methods, have recently made up a significant role in classifying and analyzing approaches for metagenomics data. But, they are challenged by various bottlenecks, such as performance limitations, high memory consumption, and computational overhead. To deal with these challenges, we developed an innovative features extraction and sequence profiling method for DNA/RNA sequences, called PC-mer, taking advantage of the physicochemical properties of nucleotides. PC-mer in comparison with the k-mer profiling methods provides a considerable memory usage reduction by a factor of 2^k^ while improving the metagenomics classification performance, for both machine learning-based and computational-based methods, at the various levels and also archives speedup more than 1000x for the training phase. Examining ML-based PC-mer on various datasets confirms that it can achieve 100% accuracy in classifying samples at the class, order, and family levels. Despite the k-mer-based classification methods, it also improves genus-level classification accuracy by more than 14% for shotgun dataset (i.e. achieves accuracy of 97.5%) and more than 5% for amplicon dataset (i.e. achieves accuracy of 98.6%). Due to these improvements, we provide two PC-mer-based tools, which can actually replace the popular k-mer-based tools: one for classifying and another for comparing metagenomics data.

## 1 Introduction

In recent decades, advances in sequencing technology have reduced the costs of genome sequencing, and consequently led to the exponential growth of genomic sequence data. Various analyses of these sequences (e.g. comparison, alignment, similarity/dissimilarity scoring) are critically important for evolutionary studies, genetic epidemiology studies, and drug design among others. Therefore, extensive efforts have been devoted to develop fast and accurate tools for analyzing and comparing genome sequences. Although most recent studies address accuracy metrics while developing classifiers for any form of DNA/RNA sequence, there exist many other crucial concerns, such as the method complexity and the resource requirements that are usually ignored in most developed approaches. Genome sequence analysis is however a tedious task because of the diversity of available datasets in terms of number of sequences, their lengths, and the degree of similarity among them [1–6]. Metagenomics data is one of most challenging form of DNA/RNA data, and their special features often lead to special requirements and assumptions for developing tools to analyze them. In this manner, the researchers aspire to achieve good performance for analyzing other forms of DNA sequences with the help of recently developed metagenomics analysis tools. This statement is true for all alignment-based and alignment-free approaches, as well as the computational and machine-learning based approaches. Therefore, we target development of an accurate metagenomics analysis tool to facilitate development of new methods analyzing various forms of DNA/RNA sequences.

Microbial communities are essential components of Earth’s various ecosystems, sustaining both environmental and human health [7–9]. Metagenomics analysis may be used in a variety of fields, including biotechnology, ecology, and bioremediation. It has also been well acknowledged for its role in improving diagnostics, as well as public health settings. Given the importance and potential applications of this information, there is a strong need to accurately profile and compare the communities they form [8]. Since metagenomics data is collected from the environmental samples, it contains an uncertain distribution of an unknown number of bacteria and viruses, some of which have never been identified previously, making sequencing and analyzing difficult [10]. Metagenomics datasets come in many forms, including marker-based datasets and whole-sequences. Specifically, the 16S rRNA gene sequence is the most extensively used marker gene for profiling bacterial communities. However other markers are needed to distinguish viruses, fungi, and other microorganisms that do not carry the 16S marker gene [10,11]. The metagenomics data itself, depending on the sequencing technology and taxonomy levels, can create a great variety in the production of datasets with different conditions of sequence similarity, which are discussed in "Data" section of S1 File. In this manner, various algorithms have been proposed so far, including computational methods and machine learning-based methods, to compare and categorize the metagenomics data. However, it should be noted that most of the studies in this field are focused on the development of classification methods with the help of machine learning-based methods onlyat the species level, although their output data can also be used for the comparison goals.

Aside from the effects of sequencing technique and its features, there are two other items which have significant impacts on the tool’s performance; a) the extracted features and the encoding methods preparing input data for the algorithms and b) the analyzing algorithms. Reads are the tool’s input in the field of metagenomics data categorization, while they are quite diverse from one another. As a result, one of the most often used encoding strategies is to use the repetition frequencies of sub-strings of fix length k, known as *k-mers*, to reduce the impact of these diversities and increase the accuracy of classification and comparison [9,11–14]. Nevertheless, one of the key challenges of the *k-mer* based methods is that they necessitate inclusion of all possible *k-mers* in the input matrix of the learning-based methods, as well as many computational methods, and this leads to the exponential growth of these matrices when the value of k rises. This is while the value of k affects the performance of data categorization and its increment improves the tool’s accuracy, from class level to genus level [11]. In all, by increasing the value of k for the *k-mer* based methods, we face the problems of high memory requirement and increased processing time, which still need to be improved.

Taxonomic binning algorithms and taxonomic profiling algorithms can be designed to be either based on alignment and assembly procedures, or alignment-free. As a main limitation of the alignment-based methods, it is worth mentioning that they only consider the identified species existing in the database, while most creatures of metagenomics samples are unknown. On the other hand, the improvement of sequencing technology and consequently growth of the database, as well as the number of samples extracted of the metagenomics data, result in huge amount of information complicating data processing and increasing memory and time consumptions [10]. Therefore, recently various methods independent of alignment and assembly procedures have been proposed and gained special importance. In fact, alignment-free methods (either computational or machine-learning-based ones) seek information from the sequences, and hence, allow different samples to be identified and differentiated at each evolutionary level. To achieve this goal, recently developed learning-based methods are adopted to extract this type of information, which is hidden in the data. Various studies, such as DBN, CNN, and RDP based methods, have shown the success of these methods [11].

Based on the above discussion, it should be noted that all aforementioned choices of data preparation strategy and the analyzing algorithms are correlated and affect the performance of the final tool. Therefore, intelligent information extraction from the sequence, to feed the algorithm, can affect both the accuracy and the complexity of the method. Thus, in developing a suitable tool for metagenomics classification or comparison, all aspects must be considered together. However, most of the studies accomplished in this area focused on the algorithm optimization. Specifically, assuming an acceptable performance of well-known encoding methods, such as *k-mer* based and word2vec methods, they assumed that improvements in various aspects of the classification tools can only be achieved by the algorithm optimization. In this way, many studies [11,15] focus on comparing different learning methods, such as NBC, RDP, RF, DBN, and CNN, on various datasets. For example, in [11], the three methods CNN, DBN, and RDP are compared considering the same *k-mer* based encoding method. Latest studies, such as those based on deep neural networks (e.g. CNN) [11,15], exhibit higher performance and appear to be capable of extracting more meaningful information when employing balanced and simulated datasets. However, some recent researches, such as [15] manipulating datasets with varying weights, show that although these approaches, validated by balanced datasets, perform better in classification, they are stuck in a rut in terms of performance development. Therefore, [15] proposes the use of auxiliary information, other than the *k-mers’* repetition frequency, to solve this performance issue. Specifically, they provide statistical data for the weight of different species within the samples, and verified the resultant performance improvement. It should be mentioned that Table 1 qualitatively compares the aforementioned methods, in terms of accuracy, complexity, and impactability based on the statistical data from unbalanced datasets.

**Table 1:** Qualitative comparison of the relative status of previous machine learning-based methods.

Classification method	Accuracy	Influenced by taxonomic weighting information	Complexity
**CNN**	High	High	High
**NBC**	Mid	Low	Low
**RF**	Mid	High	Mid
**DBN**	High	High	High
**RDP**	High	High	High

Recently, various motivations have necessitated the development of accurate, fast, and low-consumption classification methods for metagenomics sequences. The computational complexity of accurate but expensive methods, like CNN, the exponential growth of encoded data produced by various encoding methods, such as *k-mer* based methods, requirement of time-consuming training phases for some encoding methods, such as word2vec, and finally, the complexities of metagenomics data constitute the motivation of our encoding method, named PC-mer (PhysicoChemical *k-mer*). Considering the good performance of *k-mer* based approaches in metagenomics data analysis, we propose PC-mer based on the *k-mer* frequencies. On the other hand, in order to overcome the aforementioned challenges of traditional *k-mer* based encoding approaches, such as processing time and memory overhead, we propose to merge *k-mer* data with the physicochemical features of nucleotides to create a new form of *k-mer* frequency information with reduced memory usage. We examine some assessments and criteria to evaluate the capability of the PC-mer feature extraction method for various *k-mer* sizes in classifying and comparing metagenomics data. These experiments include different conditions of metagenomics data from sequencing technology to the taxonomy levels, and many reports have been prepared for them, as comprehensively explored in "Data" section of S1 File. These tests reveal that PC-mer has various incentives, such as:

1. PC-mer reduces memory consumption by about 2^k^ times in comparison to the traditional *k-mer* based encoding techniques.
2. Raising the size of k improves PC-mer classifier’s performance, which is even feasible in ordinary desktop systems due to its low memory consumption.
3. PC-mer increases classification performance for both datasets (i.e. AMP and SG), in comparison to other *k-mer* based classifier approaches [11].
4. PC-mer increases classification performance at all five taxonomic levels (i.e. Class, Order, Family, and Genus, species), in comparison to other *k-mer* based classifier approaches [11]. For example, it leads to an accuracy increase of about 14% for the genus-level classification, compared to the best alternative *k-mer* method [11].
5. PC-mer improves various classification metrics, as measured by four metrics: accuracy, F1-score, precision, and recall.
6. In comparison to the alternative *k-mer* based encoding methods, PC-mer speeds up the training phase of the classifiers [11].

## 2 Method

**Fig 1:**
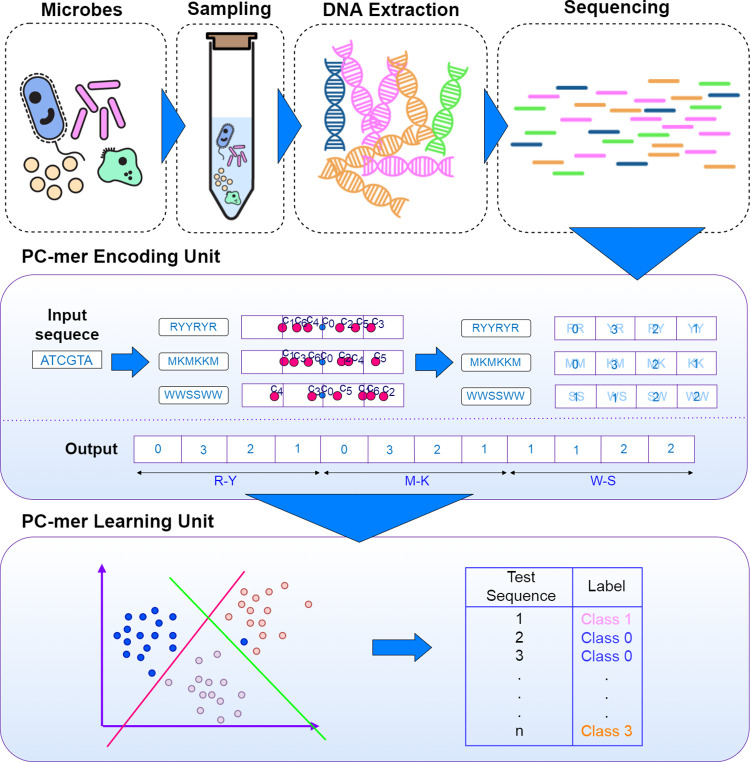
PC-mer method’s flow; preprocessing metagenomics data sequences, using them as the input of PC-mer feature extraction unit, and finally, adopting a PC-mer Learning unit to classify the sequences [3].

In this section, we explore our proposed pipeline for bacteria taxonomic classification of metagenomics data. The proposed pipeline can be divided into two main parts, as shown in Fig 1: i) a preprocessing unit generating a vector representation for each sequence to feed the machine learning-based architecture, and ii) a learning unit performing the supervised data classification. To evaluate the impact of encoding unit, we use eight popular and practical supervised-learning based classification models (i.e. Logistic Regression (LR), Gaussian naïve Bayes (GNB), linear discriminant analysis (LDA), multi-layer perceptron (MLP), decision tree (DT), Linear Support Vector Classification (SVC), nearest centroid median (NC-median), and nearest centroid mean (NC-mean)) to classify the metagenomics data. The rest of this section outlines all the steps of this process.

### 2.1 Feature extraction method

As mentioned in the previous section, in this work, we propose a novel feature extraction method utilizing the physicochemical features of the nucleotides to group *k-mers* on purpose. The PC-mer improves performance of the *k-mer* counting-based applications besides reducing their memory consumption. In this manner, considering three kinds of chemical and physical properties of the nucleotides [16], we categorize them in three sets of structural groups as follows. In the first category, *A* and *G* nucleotides are categorized as purine represented by *R*, while *C* and *T* nucleotides are pyrimidine denoted by *Y*. In the second category, *A* and *C* nucleotides are categorized as amino represented by *M*, while *G* and *T* are keto represented by *K*. And finally, in the third category, according to the H bound, *C* and *G* have strong H bond denoted by *S*, while *A* and *T* bases contain weak H bond denoted by *W*. Based on these categorizations, each pair of nucleotides can be represented by the same character in the new sets of characters. In this manner, for these new sets of characters, 2^k^ different *k-mers* exist. Concluding above discussion, the proposed conversion of character sets facilitates classification of 4^k^ classical *k-mers*, composed of nucleotides, into 2^k^ classes with 2^k^ members.

**Fig 2:**
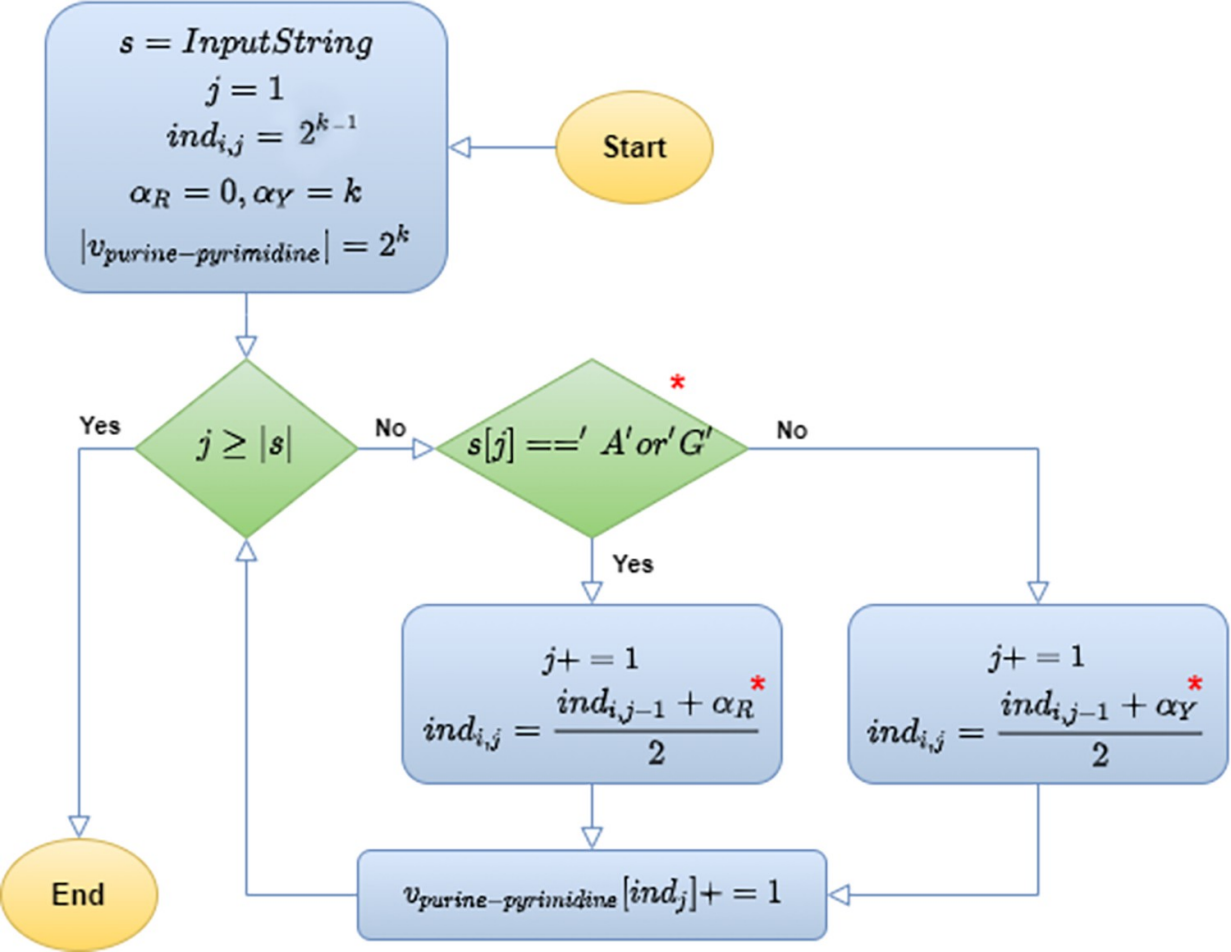
. Flowchart of PC-mer; steps of PC-mer algorithm to generate purine-pyrimidine features vector, *: It changes for each set of physicochemical properties *α*_*i*_∈{{*α*_*R*_ = 0, *α*_*Y*_ = *k*}, {*α*_*M*_ = 0, *α*_*K*_ = *k*}, {*α*_*S*_ = 0, *α*_*W*_ = *k*}}.

**Fig 3:**
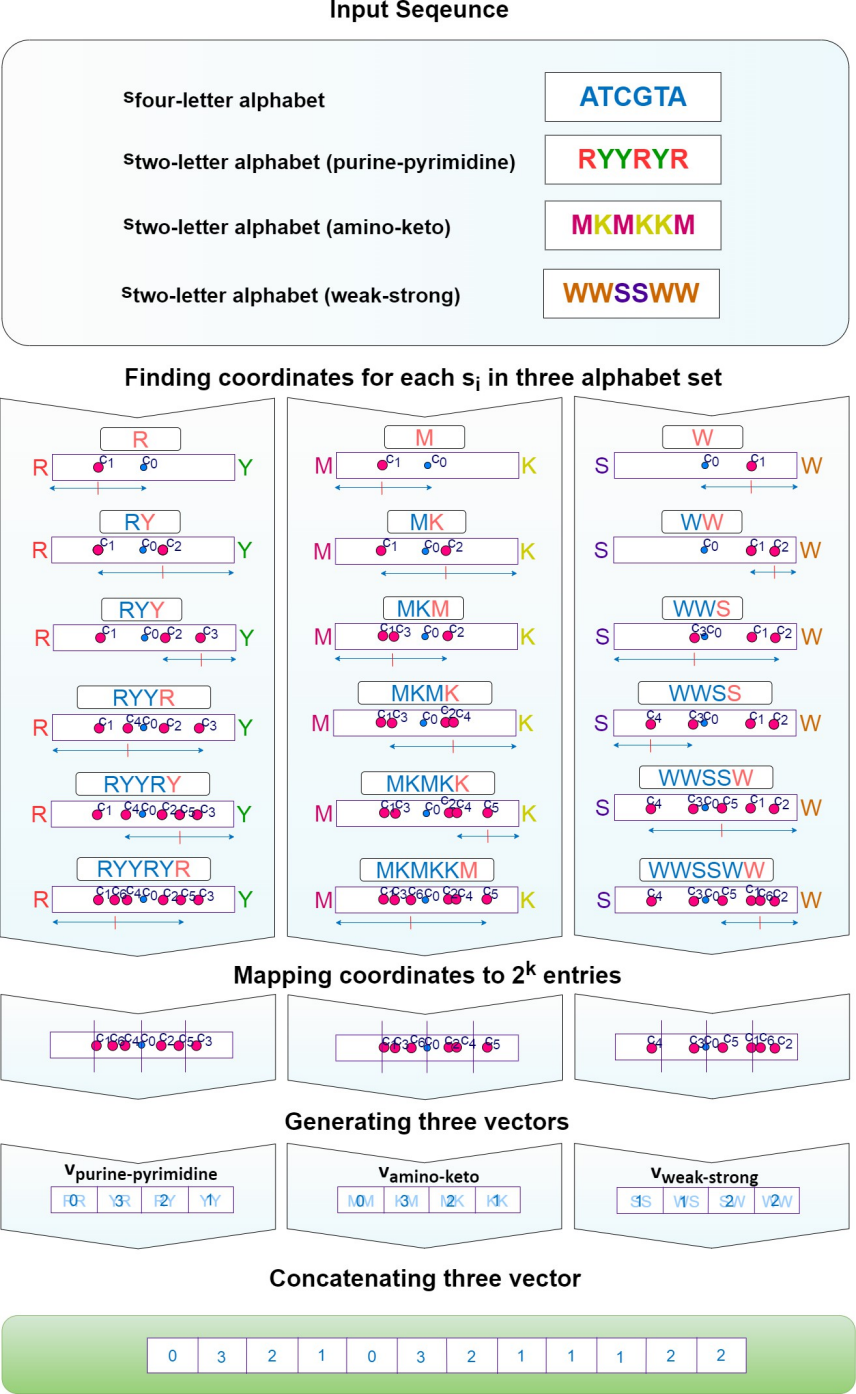
An example of PC-mer feature extraction method; steps of PC-mer algorithm to generate feature vector for sample sequence "ATCGTA" [1].

Based on the introduced character sets conversion, we propose a new DNA/RNA feature extraction method. Let α be the set of three possible pairwise compounds for nucleotides based on the chemical and physical properties, i.e. *α* = {{*R*,*Y*}, {*M*,*K*}, {*S*,*W*}}. For each entry of α, that is *α*_*i*_∈*α*, *i* = 1,2,3, we create a vector *ν*_*i*_ of length 2^*k*^, where (*α*_*i*,1_, *α*_*i*,2_), defining *α*_*i*_, is represented by two vertices of vector *ν*_*i*_. Therefore, we have three vectors, i.e. *v*_purine−pyrimidine_, *v*_amino−keto_, and *v*_*weak−strong*_, the beginning and the end of which are assigned to the *R* and *Y* as the representatives of the purine and pyrimidine categories, *M and K* as the representatives of the amino and keto, and finally, *S and W* as the substitutes for categories strong and weak, respectively. As the starting point, we select the midpoint of each vector, i.e. *v*_purine−pyrimidine_, *v*_amino−keto_, and *v*_*weak−strong*_. For each nucleotide *s*_*i*_ of a sequence *s* of length *l*_*S*_, we move forward to find the updated location (according to (1)) which is halfway between the current location and either *α*_*i*,1_ or *α*_*i*,2_, as the end points of this vector, and finally, we increase the value of the new location by one unit (according to (2)). This process, known as the Frequency of Chaos Game Representation (FCGR) algorithm, involves computing and filling an array to coordinate points. For nucleotides, we have created a new version of the FCGR algorithm by grouping them together in this work. The original FCGR algorithm is designed for the four nucleotides, resulting in an array size of 4^K^. It can be proven that each cell of the array represents the count of a specific k-mer. This holds true for our algorithm as well. In fact, each cell in our vectors, i.e. *v*_purine−pyrimidine_, *v*_amino−keto_, and *v*_*weak−strong*_ belongs to one of the *k-mer* groups, each of which has 2^k^ members. Therefore, the value of each cell represents the total number of times all members of the corresponding *k-mer* group appear in the input sequence. This process is performed for all nucleotides of s, as well as all three vectors concurrently, as shown in Fig 2. Fig 3 also depicts the PC-mer assuming a sample sequence as its input. Section "FCGR" of S1 File deals with more details about FCGR algorithm, such as its ability to count *k-mers*. Moreover, this section provides a mathematical relationship between the *k-mer* matrix traditionally generated by the four-letter character and the one generated according to our introduced character set. Finally, in Section "FCGR" of S1 File, we explore how each *k-mer* is represented by the cell’s label of the FCGR matrix, and the matrix’s fractal structure. It should be mentioned that the meaning of the fractal-like structure of FCGR is its repetitive and intricate patterns at various scales and also Its divisibility is to obtain longer K-mers. This fractal nature highlights the underlying order and complexity within DNA sequences. By analyzing the FCGR matrix, researchers can gain insights into the structural and functional properties of DNA sequences. The fractal structure of the FCGR matrix serves as a powerful tool for exploring and understanding the intricate nature of DNA.


$${ind}_{i,j}=\left\lceil \frac{ind_{i,j-1}+\alpha }{2}\right\rceil$$

$$with\;j=1,\ldots ,l_{S}\;and\;i=1,2,3\;and\;{ind}_{i,0}=(\frac{2^{k}}{2})$$

$$\alpha_{i}\in \{\{\alpha_{R}=0,\alpha_{Y}=k\},\{\alpha_{M}=0,\alpha_{K}=k\},\{\alpha_{S}=0,\alpha_{W}=k\}\}$$ (1)

$$v_{purine-pyrimidine}[ind_{1,j}]=v_{purine-pyrimidine}[ind_{1,j}]+1$$

$$v_{amino-keto}[ind_{2,j}]=v_{amino-keto}[ind_{2,j}]+1$$

$$v_{weak-strong}[ind_{3,j}]=v_{weak-strong}[ind_{3,j}]+1$$ (2)

### 2.2 Learning unit

We trained a supervised classifier based on the features extracted by our vector representation method PC-mer. It is worth noting that traditionally, existing methods use convolutional neural networks to classify metagenomics data, while taking advantage of CNN’s feature extraction and classification capabilities. However, to confirm the superiority of the proposed feature extraction scheme, we evaluated the method with eight different basic and traditional classifier algorithms. The most important reasons for adopting simple algorithms, rather than deep neural networks such as convolutional neural networks, are listed as follows:

- To avoid computational overheads of convolutional networks, in terms of time and memory requirements, while increasing the accuracy of classification
- To eliminate parameters’ adjustment, as well as development of new neural network architectures
- To enable performance evaluation of the encoding method disregarding the choice of classification algorithm
- To enable execution of the algorithm on simple computers with no customized hardware

The algorithms used for the supervised classification are: 1) logistic regression with the L2 regularization, one-vs-rest as the multiclass generalization, the stopping tolerance of 10^−4^, and the regularization strength (λ) of 1 (logistic-regression); 2) decision tree with Gini impurity metric (decision-tree); 3) Gaussian naïve Bayes (Gaussian-naive-bayes); 4) linear discriminant analysis (lda); 5) multi-layer perceptron with a single 100-neuron hidden layer, the rectified linear unit (ReLU) activation function, the Adam stochastic gradient-based weight optimizer, the L2 penalty term of 10^−4^, the learning rate of 10^−3^, and the maximum epochs of 200 (multilayer-perceptron); 6) SVC with the linear (linear-svc) and L2 penalty; 7) the nearest centroid, to class mean (nearest-centroid-mean); and finally, 8) the nearest centroid, to class median (nearest-centroid-median). The 10-fold cross-validation approach was used to evaluate the performance of all supervised classifiers. We used the implementations of these classifiers in the Python library scikit-learn, while all settings and hyperparameters were left as the default values given in the library.

## 3 Results

### 3.1 Datasets

Besides complexity of the metagenomics tools development, the lack of realistic analyzed dataset complicates validation of the classifiers, and so, defines the classifiers development as an open challenge. To resolve this limitation, continuous researches focus on the simulated dataset [11,15]. However, although the simulated datasets are widely utilized, various efforts tried to make them nearly similar to the realistic metagenomics data. In this manner, they prepare suitable datasets and benchmarks for the metagenomics classifiers by extracting realistic dataset from the popular databases, like SILVA, RDP, and QIIME [17,18]. For this purpose, various samples with different distributions among different classes are chosen and some noises are added to them. In this manner, both unbalanced and balanced datasets can be simulated. It is worth noting that while unbalanced datasets have been traditionally adopted for evaluating many metagenomics classification methods [11,15], most of the recently proposed metagenomics classifiers use balanced datasets. To address several classification scenarios, in this paper, we considered various types of datasets, as described following. One of them, we called it HTL_datasets, is the most widely used datasets discussed in [11]. It consists of 57788 16S gene sequences in the unaligned fasta format from the RDP database (version 11, update 5 dated September 30, 2016) from the bacterium kingdom, as well as the simulated reads from the shotgun and amplicon sequencing. In the first step, they created a balanced dataset at the genus level by randomly selecting a subset of these sequences from the Proteobacteria phylum, which consisted of 1000 sequences divided into 100 genera and 10 species for each genus. These 1000 sequences are then categorized into other taxonomic levels to create datasets at general levels (class, order, and family). The number of different categories belonging to each taxon is also determined as follow: 3 classes at the class level, 20 classes at the order level, and 39 classes at the family level. Of course, it is crystal clear that the numbers of samples in different evolutionary levels, except the genus level, are unbalanced. HTL_datasets are described in more details in Section “Data” of S1 File. Aforementioned datasets only encompass taxonomy levels from the class level to the genus level, while to cover the species level, we have used an unbalanced dataset introduced by [17] which is generated from Qiita database [19]. For this purpose, we have generated six datasets from the unbalanced dataset, we called them LTL_datasets, among which two of them are at the genus level and four of them are at the species level and we use them for assessing an alignment-free method based on PC-mer. More details about these datasets, such as various classes’ distribution, are discussed in Section "Data" of S1 File.

### 3.2 Overview

**Fig 4:**
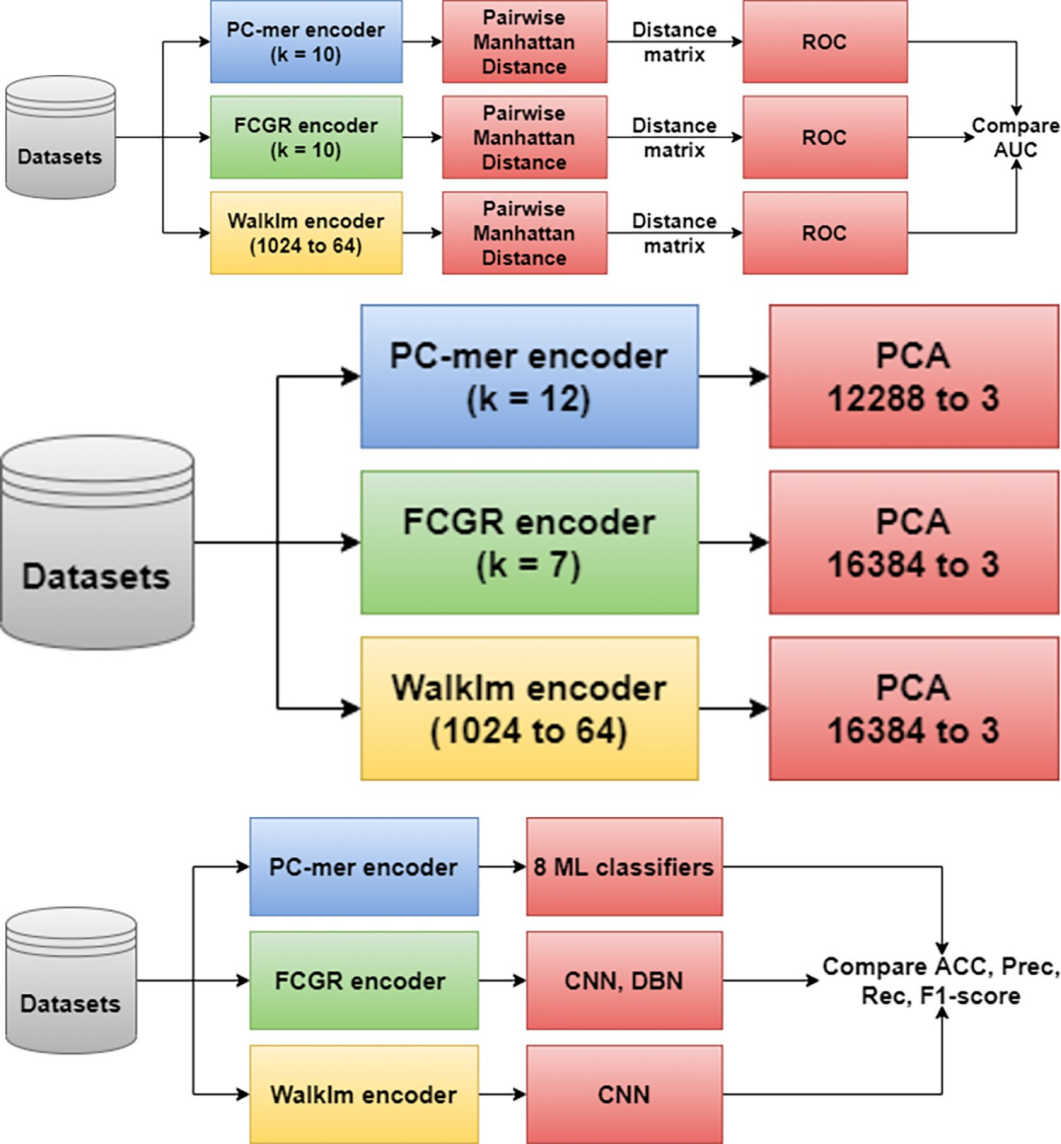
Three designed assessments clarifying key features of PC-mer feature extraction method; a) Evaluation approach comparing the computational comparison algorithm, based on Manhattan distance applied on the encoded sequences, against the three methods, PC-mer, FCGR, and WalkIm, b) PCA employment to vectors generated by the three methods, PC-mer, FCGR, and WalkIm, and c) comparing ML-based classification for three methods, PC-mer, FCGR, and WalkIm.

In this section, we evaluate the capability of the PC-mer feature extraction method and its performance as an input data generator for the classification models and comparators. For this purpose, some assessments have been considered as follows, whose steps are also shown in Fig 4:

1. Assessing the capability of PC-mer method, against a most accurate comparison method and also a traditional k-mer-based comparator, as the input generator for the sequence comparison applications, comparing various datasets from diverse taxonomic levels and identities.
2. Evaluating the capability of PC-mer method to extract appropriate features from the input sequences, compared to the state-of-art encoding methods.
3. Comparing PC-mer performance to that of the well-known encoding methods while feeding the MLP classifiers.

For the first assessment, to clarify the efficiency of the proposed encoding method, we have used Manhattan distance as the sequences’ dissimilarity score along with a well-known clustering method to compare efficiency of the encoding methods for LTL_datasets. These datasets are also chosen from various taxonomy levels and identities to evaluate the capability of PC-mer method against the state-of-art encoding methods, i.e. FCGR [11,12,20–22] and WalkIm [23]. These datasets, alongside HLT_datasets, have also been used for the second assessment. Specifically, for the second assessment, dimension reduction is applied to the preprocessed datasets using PC-mer and other state-of-the-art feature extraction methods. In this manner, we can compare capabilities of the feature extraction methods to distinguish various classes from each other. Finally, as mentioned earlier, for the third assessment, we use eight basic machine learning algorithms to classify metagenomics data. Based on ten different k values (i.e. 3 to 12), eight basic classifiers (i.e. LR, GNB, LDA, MLP, DT, SVC, NC-median, and NC-mean), and eight metagenomics datasets (four taxonomic levels of two datasets with different sequencing technologies as AMP and SG), and through a tenfold cross-validation scheme, we conducted 6400 different experiments to find the best configuration. First of all, we examine the accuracy of various architectures of machine learning-based classifiers employing the PC-mer feature extraction approach to build their input vectors for classifying various levels of metagenomics data. We then compared our best results to the best classification performances provided by the RDP classifier [7], the reference classifier for bacteria identification, WalkIm [23], and finally, CNN-DBN classifier [11]. It should be noted that all simulation results for various classifier methods have been reported in S1 File in details. Moreover, it is worth noting that the RDP, CNN and DBN metagenomics classifier methods are based on classical *k-mer* frequency encoding methods and take advantages of more features (by approximately 2^k^) than the PC-mer feature extraction method. In this manner, by referring to RDP, CNN, and DBN in the following, we mean both their classifier architecture and the feature extraction method. Afterwards, we discuss the training, testing, and preprocessing times of the best architecture we examined, and compare them against those of alternative architectures. Finally, we assess volume of the generated data, and the corresponding memory usage, as a result of PC-mer encoding method, and its advantages.

### 3.3 PC-mer in use by distance-based methods for comparison and classifying metagenomics sequences

A sequence representation method can establish a linear relationship between the sequences’ dissimilarity scores and the feature matrices’ distances. Such a feature extraction method facilitates its adoption as the input generator for both deterministic and ML-based comparison methods. Since PC-mer can be categorized as a k-mer-based feature extraction method, for the comparative study, we can examine various accurate k-mer-based sequence comparison methods, chosen from both categories of deterministic and ML-based approaches. For this purpose, we compare the PC-mer method against two different feature extraction methods; the first one is the FCGR method, which generates the *k-mer* profiles, and the second one is WalkIm encoding, which is based on DNA-walk and suitable for feeding the ML-based comparison methods. The Manhattan distance is then employed as the sequence dissimilarity metric. The result is also compared to the Smith-Waterman approach, which is known as the most accurate alignment-based approach for sequence comparison [24]. For this assessment, we employ LTL_datasets, which are appropriate for deterministic comparison approaches and provide varieties of identity, as explained in Section "Data" of S1 File. It should be noted that the k size for PC-mer and FCGR methods is assumed as 10, while the image size of 1024 × 1024 is considered for WalkIm which is scaled to 64 × 64. Finally, we evaluated the outcomes using the AUC metric for multiple classification approaches, as reported in [25], and also using the correlation coefficient between compared data extraction methods and SW for comparison approaches.

The AUC ranges from 0 to 1, with a closer value to 1 indicating a more accurate categorization strategy. AUC above 0.9 specifies a high-accuracy classifier, while AUCs between 0.7 and 0.9 and between 0.5 and 0.7 are deemed the average and low-accuracy classifiers, respectively [25]. The results for the four encoding and comparison methods are listed in Table 2. Despite having a lower number of features, compared to the k-mer-based preprocessing, the AUC values of PC-mer and FCGR are very close, while both can be specified as the high-accuracy feature extraction methods. Moreover, the similar performance of PC-mer and FCGR demonstrates that PC-mer can be used to replace the FCGR or, in general, any k-mer-based profile. In this experiment, however, WalkIm does not perform well, while its AUC values are inappropriate. Therefore, we can conclude that WalkIm cannot be adopted with the linear deterministic classification algorithms, although it is compatible with the ML-based classifier approaches. The AUC values of PC-mer and FCGR are also extremely similar to that of the Smith-Waterman, and in some cases (e.g. Acetobacter, Acidovorax, and Corynebacterium) are even higher. The latter achievement confirms that for some datasets, a k-mer-based comparison method can provide better classification performance than an alignment-based method. The latter property has been demonstrated for other types of sequence data, such as lncRNA sequences, as well [26].

**Table 2:** AUC and correlation coefficient values for three sequence representation methods (i.e. PC-mer, FCGR, and WalkIm) as the input generator for the Manhattan distance calculator as well the Smith-Waterman method.

		Genus		Species			
		Acetobacteraceae	Actinomycetaceae	Acetobacter	Acidovorax	Corynebacterium	Pyrobaculum
**AUC**	PC-mer	93.24	90.29	98.58	92.21	98.29	85.10
	FCGR	92.37	91.74	98.86	92.29	98.70	85.38
	WalkIm	64.37	54.06	74.41	86.00	85.11	65.45
	Smith-Waterman	95.54	93.88	98.42	91.39	97.90	87.86
**Corr. Coef.**	PC-mer & SW	95.21	90.91	96.67	97.09	98.58	49.33
	FCGR & SW	94.27	90.00	96.47	96.69	98.00	48.59
	WalkIm & SW	67.58	84.95	56.19	69.68	80.53	61.80

Similar behavior is also observed in correlation analysis. Specifically, to demonstrate the strong correlation between the ready-made metric utilizing PC-mer and the reference method, Smith-Waterman, we computed the correlation coefficient between their respective distance matrices. Additionally, we performed this analysis using two other methods, namely FCGR and WalkIm. This approach allows us to assess the extent to which the distance matrix derived from PC-mer can accurately determine the mutation value. According to Table 2, the PC method has the highest correlation with the reference method in most data sets compared to the other two methods.

### 3.4 Feature extraction capability of PC-mer

**Fig 5:**
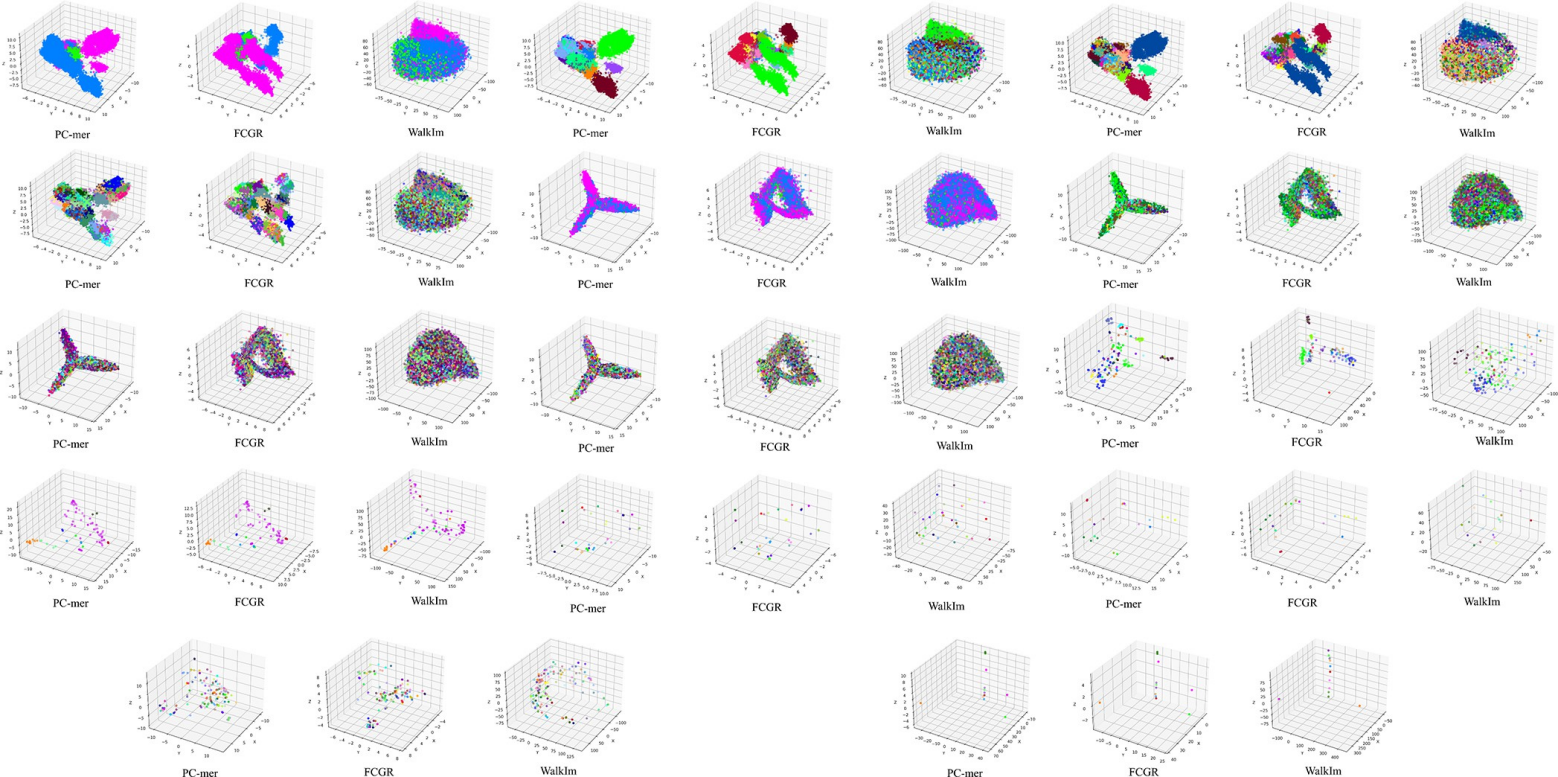
PCA of PC-mer with k = 10, FCGR with k = 10, and WalkIm with size 1024 to 65 for all datasets; a) AMP-Class, b) AMP-Order, c) AMP-Family, d) AMP-Genus, e) SG-Class, f) SG-Order, g) SG-Family, h) SG-Genus, i) **Acetobacteraceae**, j) **Actinomycetaceae**, k) **Acetobacter**, l) Acidovorax, m) Crynebacterium, n) Pyrobaculum.

While the previous section clarified the similar performance of the PC-mer and FCGR methods in sequence clustering, in this section, we analyze the quality of the extracted features from various sequences using PC-mer. For this purpose, we use the PCA approach, as a dimension reduction method, for three feature extraction methods, PC-mer, FCGR, and WalkIm, and generate a 3D plot for each dataset. As a result, we can demonstrate the clustering capability of each encoding method correspondingly. Of course, as the main goal of this analysis, we compare PC-mer and FCGR methods in terms of feature extraction capability, as can be achieved by the 3D plots. As shown in Fig 5, in most datasets, PC-mer and FCGR methods create the same 3D PCA plots. This study also demonstrates that PC-mer and FCGR methods, compared to the WalkIm, can clearly distinguish various samples, and so, suggests that simple classifiers based on the feature distance can achieve accurate results when fed by either PC-mer or FCGR method, while WalkIm representation method should be joined with the complex classifiers, like neural network-based methods.

### 3.5 PC-mer in use by the metagenomics ML-based classifier

The existence of a linear relationship between the sequence similarity/dissimilarity and the distance of the feature matrices produced by the PC-mer method, as well as its similar performance to the *k-mer* based methods have been investigated in the previous two sections. These studies lead to the conclusion that PC-mer can replace 16s k-mer-based classifier and comparator methods, and achieve equal or better classification performance. On the other hand, since learning methods based on the input sample distance are usually considered as the simple classifier approaches, in this section, we evaluate the feature extraction efficiency of the PC-mer feeding an MLP classifier.

#### 3.5.1 Training and testing procedure

**Fig 6:**
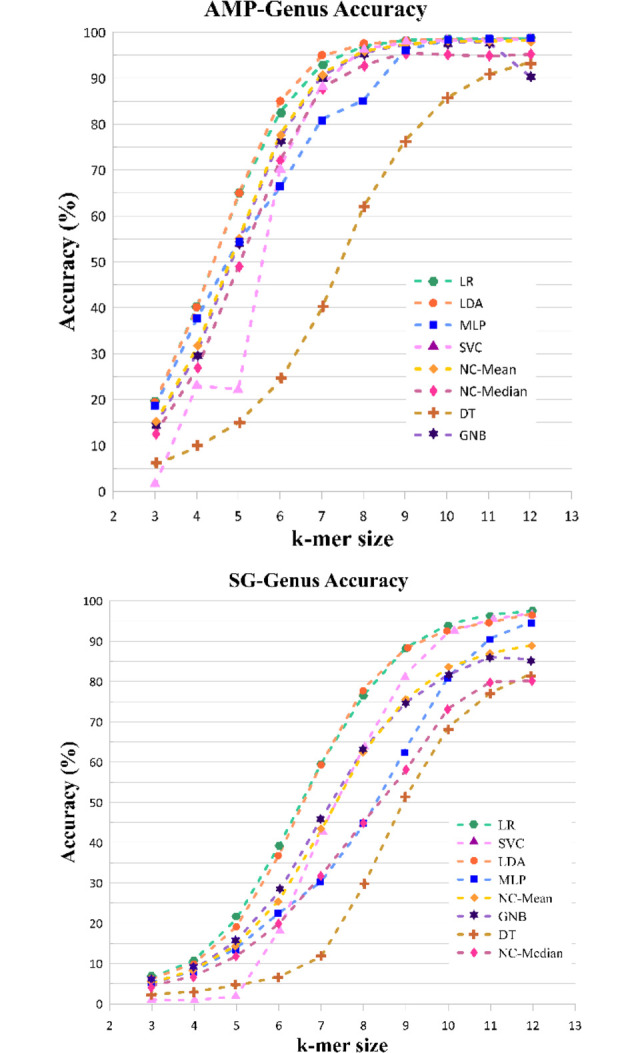
Comparing the accuracy of eight different machine learning-based classifiers employing the PC-mer feature extraction approach with size k in the range of [3,12]; a) AMP genus level, b) SG genus level.

Using eight machine learning algorithms at different levels of metagenomics data, we evaluated the performance of the proposed feature extraction method. It is worth noting that adoption of the PC-mer feature extraction method eliminates initialization of network parameters and network’s design optimization. The examined classification algorithms are listed as LR, GNB, LDA, MLP, DT, SVC, NC-median, and NC-mean. All parameters were left at their default values, and no adjustment was performed. We evaluated the PC-mer performance by its feature extraction capability and the way its performance is affected by varying the *k-mer* size from 3 to 12. Classification performances have been evaluated using the most used statistical measures, such as accuracy, precision, recall, and F1-score. According to our comprehensive simulation studies, the logistic regression classification algorithm, adopting the PC-mer feature extraction method, is the most effective one in classifying two types of input data, SG and AMP data. The part of trends of statistical measure scores for metagenomics data classification using the logistic regression, at each taxonomic level, are presented in Table B in S1 File. The results of all cases are reported in S1 File. Table B in S1 File represents the accuracy changes as *k-mer* size varies for two input dataset types. As shown in this table, the highest accuracy scores are reached with the largest value of *k-mer* size, that is k = 12. For k = 12, accuracy scores range from 100% at the taxon level to about 97% at the genus level. These results will be further discussed in the next section. It is worth noting that at the genus level, 100 different categories complicate the classification task. In this regard we present detailed results at the genus level for all eight classification algorithms for different values of *k-mer* size, as shown in Fig 6.

In Table 3, we show the superiority of our method over reference methods, like RDP, CNN-DBN, and WalkIm, for classifying metagenomics data (the results of all k size are reported in S1 File). It should be noted that WalkIm approach has not been tuned for metagenomics data, and also, it has only examined AMP sequencing technology datasets. As shown in this table, at the genus level, the accuracy of PC-mer method and the corresponding classification algorithm is significantly higher than that of other three methods. Specifically, for the AMP and SG datasets, we improved the genus level classification accuracy from 91.37 and 85.50 obtained by CNN-DBN to 98.63 and 97.5 using the PC-mer method, respectively. It is worth noting that classification results for two input dataset, 10 values of *k-mer* size, and 8 different classification algorithms are summarized in S1 File. According to confusion matrices, input data from various categories are well predicted and false negative and the false positive rate is drastically reduced. It is also important to acknowledge that the PC-mer method does not consistently outperform K-mer-based methods across all values of K. However, it excels beyond a certain value of K. Specifically, this trend is observed in the case of the AMP dataset starting from a K value of 7, and in the SG dataset starting from a K value of 9. Our experimental evaluation involved assessing K-mer-based methods up to K = 7, while we evaluated the PC-mer method up to K = 12. This difference in evaluation range was determined by considering the number of features. We aimed to increase the value of K for PC-mer to a level where its total number of features remained lower than that of the K-mer-based methods with K = 7. Consequently, we limited our testing to a maximum K value of 12 (number of features for PC-mer method with K = 12 is 2^12^), as the features of PC-mer were still fewer compared to the K-mer-based method with K = 7 (number of features for K-mer-based method with K = 7 is 4^7^ = 2^14^).

**Table 3:** Evaluating AMP and SG datasets classification at the genus level utilizing LR classifier and PC-mer feature extraction method.

Datasets	Algorithm	k	Accuracy (%)	Precision (%)	Recall (%)	F1-score (%)
AMP	CNN	3	51.01	51.40	50.90	50.84
		4	77.69	77.91	77.69	77.57
		5	88.38	88.07	88.07	88.98
		6	90.92	91.14	90.91	90.82
		7	91.33	91.57	91.32	91.18
	DBN	3	56.69	57.88	56.62	55.56
		4	85.10	85.47	85.08	84.53
		5	89.82	90.12	89.82	89.63
		6	90.55	90.37	90.53	90.45
		7	91.37	91.62	91.37	91.26
	RDP	-	83.84	84.42	83.57	83.65
	PC-mer + LR	3	19.28	17.21	19.28	17.42
		4	40.09	39.21	40.09	39.12
		5	64.37	64.42	64.37	64.08
		6	82.62	82.94	82.62	82.57
		7	**93.08**	**93.26**	**93.08**	**93.08**
		8	**96.95**	**97.03**	**96.95**	**96.95**
		9	**98.28**	**98.31**	**98.28**	**98.28**
		10	**98.52**	**98.53**	**98.52**	**98.51**
		11	**98.60**	**98.61**	**98.60**	**98.60**
		12	**98.63**	**98.65**	**98.63**	**98.62**
	WalkIm	-	98.55	89.17	89.03	89.05
SG	CNN	3	17.02	17.32	16.53	16.69
		4	32.98	33.42	32.59	32.65
		5	59.80	60.34	59.41	59.31
		6	80.77	81.10	80.41	80.33
		7	85.50	85.70	85.20	85.11
	DBN	3	17.75	19.80	17.50	16.32
		4	54.11	55.62	53.67	53.17
		5	71.44	72.45	71.07	70.99
		6	77.85	78.36	77.53	77.47
		7	81.27	81.87	80.92	80.94
	RDP	-	80.38	80.83	80.18	80.09
	PC-mer + LR	3	06.49	05.28	06.49	05.09
		4	10.80	09.33	10.80	09.47
		5	21.12	19.99	21.12	20.12
		6	39.14	38.64	39.14	38.51
		7	59.61	60.08	59.61	59.48
		8	76.59	77.24	76.59	76.64
		9	**88.39**	**88.66**	**88.39**	**88.39**
		10	**94.22**	**94.34**	**94.22**	**94.19**
		11	**96.61**	**96.69**	**96.61**	**96.58**
		12	**97.50**	**97.57**	**97.50**	**97.48**

#### 3.5.2 Execution times

As another advantage of our proposed feature extraction method, PC-mer, it can significantly improve the total processing time, which includes the preprocessing, training, and testing times. It should be noted that this speed up, compared to alternative methods, is achieved for every size of *k-mer*. Thanks to its powerful feature extraction algorithm and thus, facilitating usage of simple machine learning models to classify metagenomics data, all classification algorithms, in preprocessing, training, and testing steps, have been performed on a desktop computer and a CPU processor. However, for executing method CNN-DBN [11], a cluster with 24 nodes is utilized. Table 4 shows the execution time comparison for the training and testing steps for the logistic regression, as the best classification algorithm according to our simulation studies. As a key superiority of the PC-mer method, we can present its very small preprocessing time for data preparation. Although data preprocessing is done only once, this issue is addressed in many researches as well. In this regard, the preprocessing time for classifying different levels of metagenomics data is shown in Table 4. All experiments were performed on a normal desktop computer (CPU: i7-6500 2.5 GHz, RAM: 8 GB RAM, HD: 256GB Lexar, GPU: GeForce GTX 920M, and OS: Microsoft windows 10).

**Table 4:** The runtime of the training and testing phase of three methods.

k	PC-mer preprocessing and LR			DBN		CNN		FCGR preprocessing (DBN & CNN)
	Train (s)	Test(s)	PC-mer method	Train (s)	Test(s)	Train (s)	Test(s)	
3	17.518	0.003	0.0029	7288.913	0.111	686.403	0.240	0.0129
4	18.331	0.004	0.0029	8170.077	0.122	1256.652	0.375	0.0129
5	19.236	0.005	0.0029	11875.716	0.060	3091.721	0.719	0.0129
6	23.684	0.008	0.0029	20346.112	0.053	8021.737	1.506	0.0129
7	31.869	0.015	0.0039	37161.237	0.128	24204.754	3.986	0.0435
8	42.865	0.046	0.0039	---	---	---	---	0.0435
9	59.384	0.056	0.0042	---	---	---	---	0.0576
10	124.255	0.140	0.0042	---	---	---	---	0.0981
11	216.673	0.348	0.0049	---	---	---	---	0.1278
12	2140.572	0.811	0.0049	---	---	---	---	0.3221

### 3.6 Memory usage

**Fig 7:**
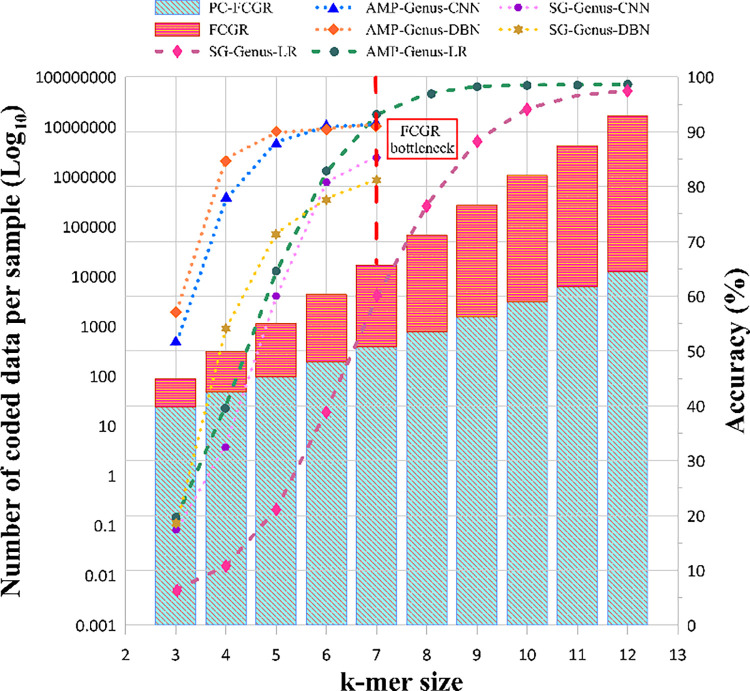
Best-case comparison of different classifiers for two sequencing technologies at the genus level for different k values along with the amount of memory usage for the two feature extraction methods, FCGR and PC-mer.

Memory consumption is one of the main limiting factors preventing usage of large size *k-mers* in k-mer-based methods. The computational overhead of k-mer-based methods, like FCGR, is very high and is defined as *O*(4^*k*^), which slows down processing step and leads to large volumes of vectors. Accordingly, we have to set a threshold value for the *k-mer* sizes, which on the other hand, leads to accuracy reduction of the input data classification. To resolve this limiting issue, our encoding method, PC-mer, is designed to reduce the computational overhead of *k-mers*, as well as the volume of the generated data from *O*(4^*k*^) to *O*(3×2^*k*^). In this manner, PC-mer allows usage of larger *k-mers* by reducing the size of encoded data. For example, assuming *k* = 7 in FCGR method, a vector of size 16,384 is generated for each genome sequence, while in PC-mer, for each genomic sequence, with *k* = 12, a vector of size 12,288 is generated which is much smaller than that of k = 7 in FCGR. It should be mentioned that assuming *k* = 12 in the FCGR method, a vector of size 16,777,216 is produced per sample. For a comprehensive comparison, Fig 7 depicts the trend of volume increment of the preprocessed data for each genomic sequence using PC-mer method, compared to the FCGR method for 3≤*k*≤12.

## 4. Discussion

As discussed in the previous sections, metagenomic analysis is increasingly becoming an important focus for the scientific community; it enables the characterization of bacterial communities derived from a particular environment without the use of cell cultures. However, it is challenging to identify the correct composition of metagenomics data from their reads with any kind of sequencing technologies (AMP and SG). Of course, AMP Miseq and SG Illumina Hiseq have a number of similarities and differences as well. For example, the SG Illumina Hiseq technology requires a longer runtime and is more expensive. On the other hand, there is a potential for using AMP in metagenomics profiling investigations when there is a lack of input material or high-speed processing is required.

As discussed above, *k-mer* profiling method is one of the most well-known encoding algorithms and has demonstrated excellent performance over the last few years, particularly in the classification of labeled data [11,20]. Nevertheless, one of its greatest challenges is the large volumes of extracted data per sample. In addition this issue is manifest in the areas of classification accuracy, training speed, and memory consumption. Recently, to overcome accuracy reduction of the preprocessed data classification, deep neural networks, which have shown remarkable performance in extracting hidden features of data and classifying them, are being considered. However, the computational overhead of deep neural networks and requiring adjustment for each dataset might limit their applicability. For example, [27] performed several experiments to adjust the size and the number of kernels of the adopted convolutional networks to achieve the best classification accuracy. Of course, most studies suggested powerful processing platforms to solve this problem [11], but this solution is not the best and they are expensive and not available to everyone. On the other hand, Data transfer to the public servers also poses the problem of compromising data security, which in turn is crucial for the biologists.

To deal with these drawbacks, we introduce a new efficient k-mer-based feature extraction method, capable of executing on desktop computers. As described in “Feature extraction method” section, our proposed feature extraction algorithm, named PC-mer, reduces the volume of feature vectors generated per sample from *O*(4^*k*^) to *O*(3×2^*k*^). The proposed method enables us to use larger values of k while still producing less data, compared to the traditional *k-mer* profile. Utilizing PC-mer can be helpful in several ways. Firstly, it leads to higher accuracy of data classification using basic classification algorithms (deterministic and ML-based), with no prior adjustments. Secondly, it greatly reduces the memory usage, and eventually, the speed of training is increased dramatically. On the other hand, it has significantly reduced false negative rates at various taxonomic levels. In addition to its compatibility with the ML-based approaches, due to the linear relationship between the sequence similarity/dissimilarity and the distance of the feature matrices produced by the PC-mer method, as discussed in Section 2.3, it can also be adopted in the computational comparison methods.

For more details, our comprehensive studies, based on 6400 experiments on 8 levels of metagenomics data (i.e. 4 levels for AMP and 4 levels for SG), 8 basic machine learning algorithms, 10 values of k, and 10-fold cross-validation approach, we can conclude that the proposed feature extraction method performs better than the alternative methods [7,11]. Indeed, the suggested classifier achieves a 100% accuracy in classifying samples at the class, order, and family levels, according to our detailed simulation analysis. It also increases genus level classification accuracy by more than 14% for the shotgun dataset (achieving 97.5% classification accuracy) and more than 5% for the amplicon dataset (i.e. achieves classification accuracy of 98.6%). These accuracy improvements for 3 evaluation scenarios, including the usage of CNN and DBN with FCGR and the usage of LR with PC-mer method, are shown in Fig 7. Another point that can be taken from this figure is that with increasing the value of k, the accuracy increases at all taxonomy levels. Specifically, from the values of k equal to 7 and 9 for AMP and SG data, respectively, the performance of PC-mer overtakes other methods. Moreover, this figure compares the amount of memory consumption for different encoding approaches. Finally, PC-mer, compared to the alternative methods, obtains an excellent speedup of more than 1000x for the training phase and around 100x for the preprocessing phase producing the input vectors. It is worth noting that we achieved this speedup by a simple CPU processor, while CNN-DBN has used a processing cluster of 24 nodes [11].

## Supporting information

S1 File All supplementary data are available at this file. (DOCX)
