# Supplementary material for: PC-mer: An Ultra-fast memory-efficient tool for metagenomics profiling and classification
Source: PLoS One. 2024 Aug 1;19(8):e0307279. doi: 10.1371/journal.pone.0307279 (PMC11293629; doi:10.1371/journal.pone.0307279)
Supplement: S1 File — (DOCX) [file pone.0307279.s001.docx]

**PC-mer: An Ultra-fast memory-efficient tool for metagenomics profiling and classification**

**Saeedeh Akbari Rokn Abadi^1^ – Amirhossein Mohammadi^1^ – Somayyeh Koohi^1,*^**

^1^ Department of Computer Engineering, Sharif University of Technology, Tehran, P.O. Box: 11155-9517, Iran

^*^ Correspondence, E-mail: koohi@sharif.edu

Contents

[FCGR 2](#_Toc114046536)

[General form of FCGR algorithm 2](#_Toc114046537)

[FCGR features 3](#_Toc114046538)

[PC-MER vs. FCGR 4](#_Toc114046539)

[Data and assessments conditions 6](#_Toc114046540)

[Investigating the impact of size k in the PC-mer method 8](#_Toc114046541)

[Evaluating classification at the genus level utilizing LR classifier and PC-mer method 17](#_Toc114046542)

[Confusion matrices of utilizing LR classifier and PC-mer method 18](#_Toc114046543)

[References 24](#_Toc114046544)

**Note:** Our comprehensive studies are based on 6400 experiments on 8 levels of metagenomics data (i.e. 4 levels for AMP and 4 levels for SG), 8 basic machine learning algorithms, 10 values of k, and 10-fold cross-validation approach. Selected and important simulation results are included in this document. To access other results contact the authors by email.

# FCGR

## General form of FCGR algorithm

In many genomic sequence classification studies, a feature extraction method called Chaos Game Representation (CGR) is used to represent the sequences. CGR is an iterative mapping technique based on Markov probability table that processes sequences of units, such as nucleotides in a DNA sequence or amino acids in a protein, in order to find the coordinates of their position in a continuous space. CGR space produced by a sequence, with $n$ unique alphabets, is a polygon bounded by *n* possible alphabets. Specifically, for genomic sequences, CGR space is a plane bounded by four possible nucleotides (i.e. Adenine (A), Thymine (T), Cytosine (C), and Guanine (G)) as vertices of a binary square. Rather than arranging nucleotides in a linear way, CGR, as a novel holistic approach, provides a visual representation of a DNA sequence. The CGR position $CGR_{i}$ of each alphabet $s_{i}$ of a sequence $s$ of length $l_{S}$ is calculated by moving a pointer to half the distance between the previous position and the current binary representation (Eq. S 1). For DNA sequences, the binary square CGR vertices are assigned to the four nucleotides as A = lower left (0, 0), T = lower right (0, 1), G = upper right (1, 1), and C = upper left (1, 0). The procedure is illustrated in Figure A.

| ${CGR}_{i}=0.5 . \left( {CGR}_{i-1}+P \right)$  $\mathrm{with} i=1,\ldots, \left\vert s \right\vert and {CGR}_{0}=\left( 0.5,0.5 \right) and P\in\{A,C,G,T\}$ | Eq. S 1 |
| --- | --- |

CGR extracts important information from the number of substrings occurrences with length $k$, named *k-mer*. Each *k-mer* defines a coordinate space in a $4^{k}$ dimensions vector space, assuming k-mers of length $k$, and four possible values for each spot (A, C, G, T). As proven, the abundance of all k-mers (oligonucleotides of length k) in a given sequence can be determined by dividing the CGR space with a grid of appropriate size and counting k-mer’s occurrences in each quadrant. In order to obtain the frequency matrix of oligonucleotides of length $k$, a $2^{k}\times2^{k}$ grid must be used and this largely covers all $4^{k}$ possible k-mers. This matrix is called FCGR (Frequency of CGR). Figure Adepicts an example of FCGR generation from a sample sequence.


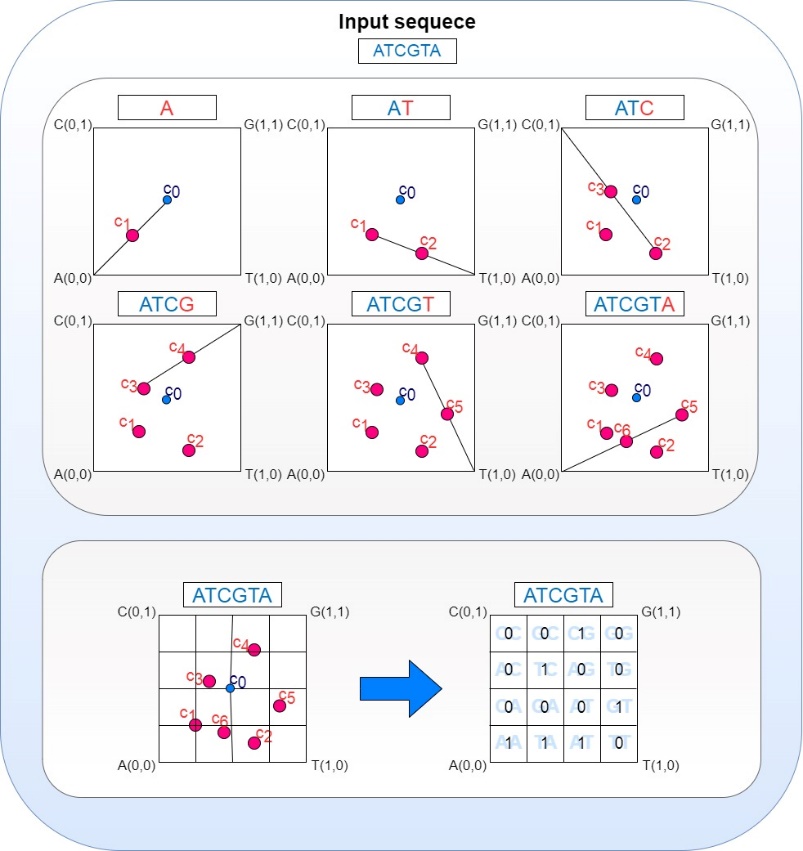


Figure A CGR and FCGR methdo

## FCGR features

As described in the previous section, and based on an earlier study described in [1], the original FCGR algorithm generates two-dimensional images to represent various patterns in the DNA sequences. Specifically, assuming the FCGR algorithm generates pixels with a granularity of 2^k^ , for a positive integer k, Jeffrey [1] demonstrated that each pixel corresponds to a distinct character string of length k [1][2]. Essentially, the whole collection of words frequencies, discovered in a given genomic sequence, can be shown as a single image, with each pixel representing a different word. While the word frequency within the sequence is shown in a square graphic, its placement is determined by a recursive method, as described following. The image is split into four quadrants, each of which collects sequences that terminate with the proper base. A simple argument can be used to demonstrate FCGR's capacity in k-mer counting, as well as its other key properties. Since the FCGR algorithm is a recursive one, we consider a square space for a set of four-letter alphabets. By reading the first letter of the input sequence, the coordinates of its point are placed in one of four subsquare areas generated by halving the sides, according to the FCGR algorithm. In other words, each of these square areas is assigned to one of the alphabet letters, as marked at the closet corner to them. This assignment is depicted in Figure B - a. These new square regions now resemble the original square space, in which the four letters of the alphabet are similarly arranged at the four corners. The second letter of the sequence is inserted into new subsquares created by halving the sides of the current subsquare. As a result, new square regions are created that belong to the two-letter subsequences. To identify k subsequences in the two-dimensional area, this happens recursively by halving the sides of the initial square k times. Figure B - b shows this process for three consequent steps.

| 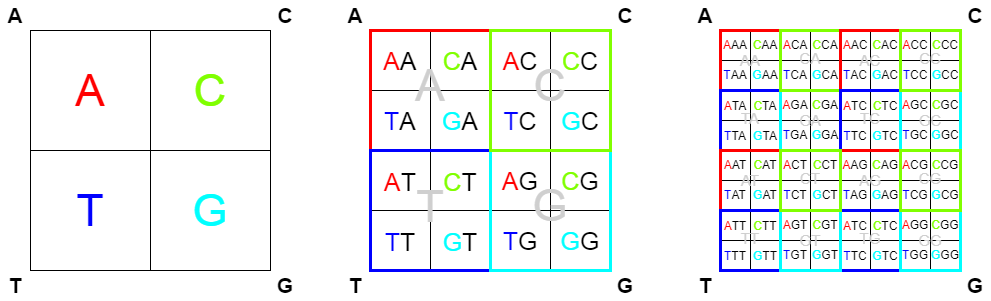 | 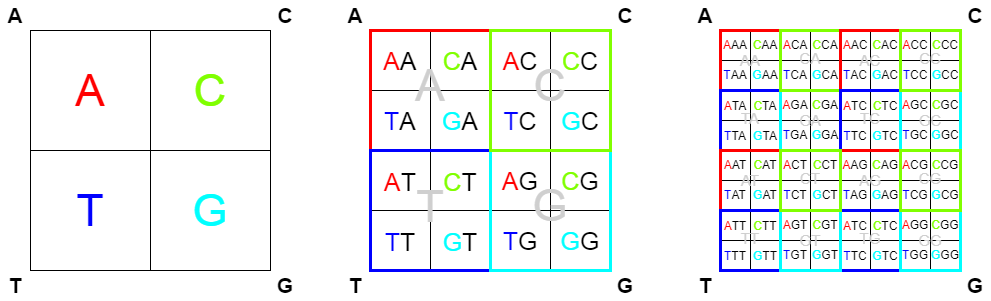 | 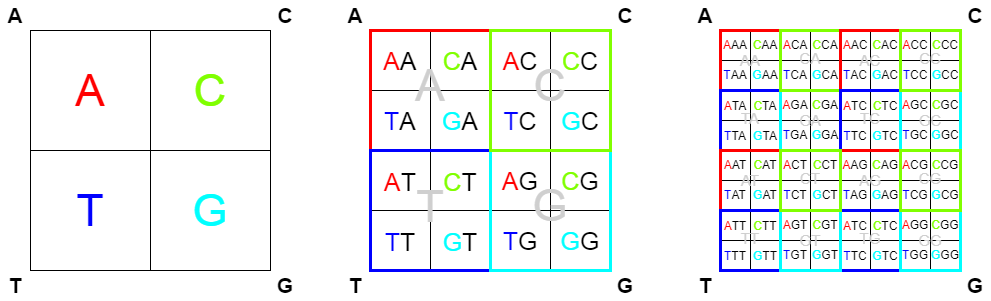 |
| --- | --- | --- |
| **a** | **b** | **c** |

Figure B FCGR fractal structure and how to label its entries; a) k = 1, b) k = 2, and c) k = 3

Furthermore, since a base is plotted in its corresponding quadrant, the converse is true: if two points are located within the same quadrant, they correspond to the sequences with the same last base; and so on, if they are in the same sub-quadrant, they correspond to the sequences with the same last two bases; if they are in the same sub-sub-quadrant, they correspond to the sequences with the same last three bases, and so on. As a result, based on this induction, we can prove the proposition that PC-mer can count k-mers' occurrence of a sequence [1]. Two sequences with suffixes of length k are contained within the square with side length 2^k^ within a CGR whose side is of length unit. Furthermore, the following recursive definition yields the square's center:

(a) The center of the zero-length suffix is (1/2, 1/2).

(b) Assuming (x, y) as the center of the square containing sequences with the suffix w:

1. Center of the square containing sequences with suffix wA is $\left( \frac{x}{2},\frac{y}{2} \right)$

2. Center of the square containing sequences with suffix wT (or wU) is $\left( \frac{x}{2},\frac{y+1}{2} \right)$

3. Center of the square containing sequences with suffix wG is $\left( \frac{x+1}{2},\frac{y+1}{2} \right)$

4. Center of the square containing sequences with suffix wC is $\left( \frac{x+1}{2},\frac{y}{2} \right)$

For each aforementioned case, all points located within the square, represent the sequences with the specified suffix.

## PC-MER vs. FCGR

As explored in details in the manuscript, PC-mer is a feature extraction method based on k-mer that maps sequences of different lengths into one dimensional vector using two letter alphabets. Specifically, PC-mer vectors have a fractal structure, while the labels assigned to each entry is derived similar to the FCGR algorithm, but with two alphabets, as explored in Section “FCGR features”. It is worth noting that based on its feature extraction algorithm, PC-mer’s output is related to that of the FCGR, as discussed following.

In the square space of FCGR, which actually belongs to the four-alphabets set of nucleotides, the alphabets assigned to the corners can be grouped together in three vertical, horizontal, and diagonal directions to form groups of physicochemical properties, as defined by PC-mer. Now, without losing the whole issue, we consider Amino (M) and Keto (K) category, and also consider the position of the alphabets as shown in Figure C-a. Thus, in the input sequence, whether A or C is observed, the upper region of the square matrix is ​​selected, and whether G or T is observed, the lower region of the square matrix is ​​selected. In this manner, by traversing the letters of the input sequence, the above algorithm is repeated in sub-regions. Based on this algorithm, it seems that PC-mer adds up the values ​​of each column of the square matrix, and so, a single row is generated from the initial matrix to represent the Amino and Keto, due to fact that the labels in the row are actually the letters A and C (Amino) together, as well as the letters T and G (Keto) together, and they have produced distinct combinations in fixed locations. A similar algorithm is performed for the other two nucleotide categorizations. The $v_{weak-strong}$ is generated by adding up the values ​​of each column of the square matrix, as shown in Figure C-b. Finally, for {R, W} grouping, diagonal patterns are created which consist of combinations of the letters A and G together, and the letters C and T together, as shown in Figure C-c.

| 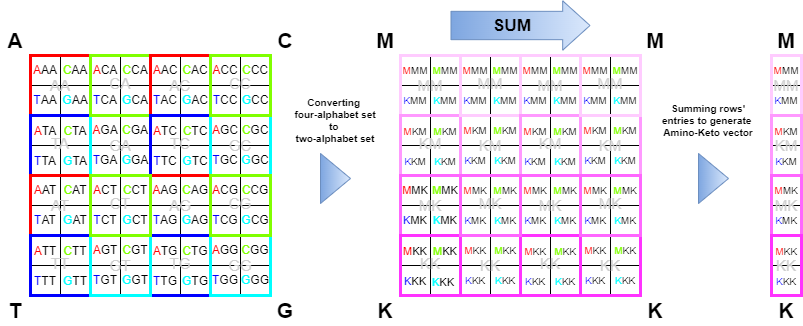 |
| --- |
| **a** |
| 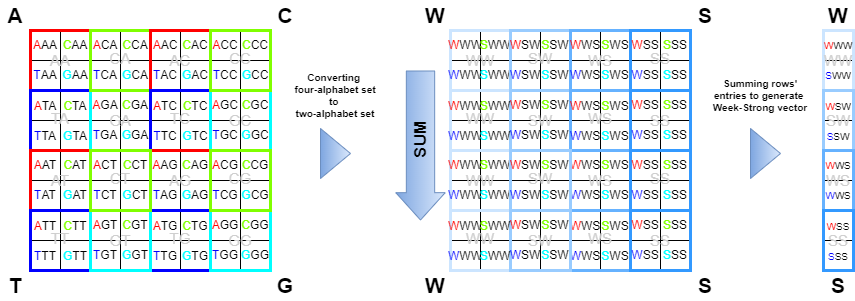 |
| **b** |
| 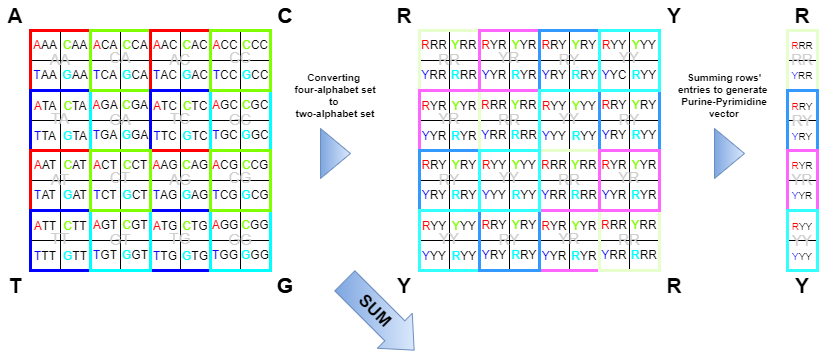 |
| **c** |

Figure C Converting FCGR to PC-mer for k = 8

# Data and assessments conditions

Two separate NGS methods are used for metagenomics sequencing [5]: a) whole genome shotgun (WGS) sequencing technique; which permits all bacterial genes to be sequenced, and b) amplicon sequencing (AMP) technique; which only sequences specific parts of the nine hypervariable areas of the 16S gene. Both approaches have various advantages and disadvantages: shotgun (SG) Illumina Hiseq technology, for example, has a greater instrument cost and a longer runtime, compared to the AMP Miseq methodology. As a result, AMP has the potential to be used in metagenomics profiling investigations when speed or a limited amount of input material is the issue. Additionally, in comparison to the SG approach, AMP sequencing provides a more thorough detection of uncommon species in complex populations. Sequencing and analysing AMP, on the other hand, has certain technological restrictions, such as chimaera production during the PCR stage and sequencing errors. However, it should be mentioned that both approaches take advantage of high accuracy (more than Q30), a read length of up to 150 base pairs (bp), and a DNA concentration of 50-1000 nanograms (ng) [5].

Another aspect to consider in studying metagenomics data is the taxonomic level of the classes under consideration, which are divided into eight categories: Domain, Kingdom, Phylum, Class, Order, Family, Genus, and Species Identifier. As these levels move closer to the identifier level, samples become more and more similar to one another, making it more difficult to distinguish various labels of each sample. It is worth mentioning that although the genus level is a popular and crucial level for categorization, it also has a lot of accuracy issues. For this reason, it has been focused in most studies.

We used two different datasets for various experiments in this study. One of these, HTL_datasets, is the most often used dataset in the presentation [3]. A pre-labeled dataset with the taxonomy of each read is required to thoroughly train and validate the proposed classification algorithms. Since reads in public metagenomic datasets lack a taxonomic classification, [3] developed a simulated dataset by generating metagenomic reads using the approach outlined in [4][5]. Because certain tools, such as REAGO [4], can recognize reads belonging (or not) to 16S with near-perfect accuracy (99%), they produced short-reads belonging to 16S (rather than the WGS). [3] used known metagenomic analysis tools to simulate readings from shotgun and amplicon sequencing. They retrieved a set of 16S gene sequences from the RDP database (release 11, update 5 dated September 30, 2016) that belonged to the bacterium kingdom in unaligned fasta format. The data were then filtered using the following parameters: Type and non-type strains; isolates as a source; size higher than or equal to 1200; Good quality. They were able to get 57788 16S gene sequences as a consequence of their efforts. They used a random subset of these sequences belonging to the Proteobacteria phylum, which consisted of 1000 sequences with 100 genera and 10 species of each genus, to create a balanced dataset at the genus level.

Finally, to evaluate the linear relationship between the input sequence and the generated PC-mer at the species level, another set of datasets, LTL_datasets, was used in this study. LTL_datasets contains six datasets which are selected from test dataset of [6]; two of which are at the genus level, while four of which are at the species level. Since these datasets have been aligned using the Smith-watterman algorithm, identities between their samples have been determined, as shown in Table A. Moreover, Figure D shows identity distribution diagrams of the mentioned six datasets, Acetobacteraceae, Actinomycetaceae,Acetobacter, Acidovorax,Corynebacterium, and Pyrobaculum.

Table A Minimum, maximum, mean, and standard deviation of identity score calculated for aligned samples by the smith-waterman method for LTL_datasets

|  | | Genus | | Species | | | |
| --- | --- | --- | --- | --- | --- | --- | --- |
|  |  | Acetobacteraceae | Actinomycetaceae | Acetobacter | Acidovorax | Corynebacterium | Pyrobaculum |
| Seq. Identity | Min. | 24.76 | 29.21 | 30.15 | 37.11 | 22.87 | 25.58 |
|  | Max. | 100 | 100 | 100 | 100 | 100 | 100 |
|  | Mean | 89.44 | 42.92 | 92.32 | 93.85 | 49.20 | 71.14 |
|  | Std. Dev. | 13.41 | 14.68 | 15.95 | 14.52 | 21.19 | 33.80 |


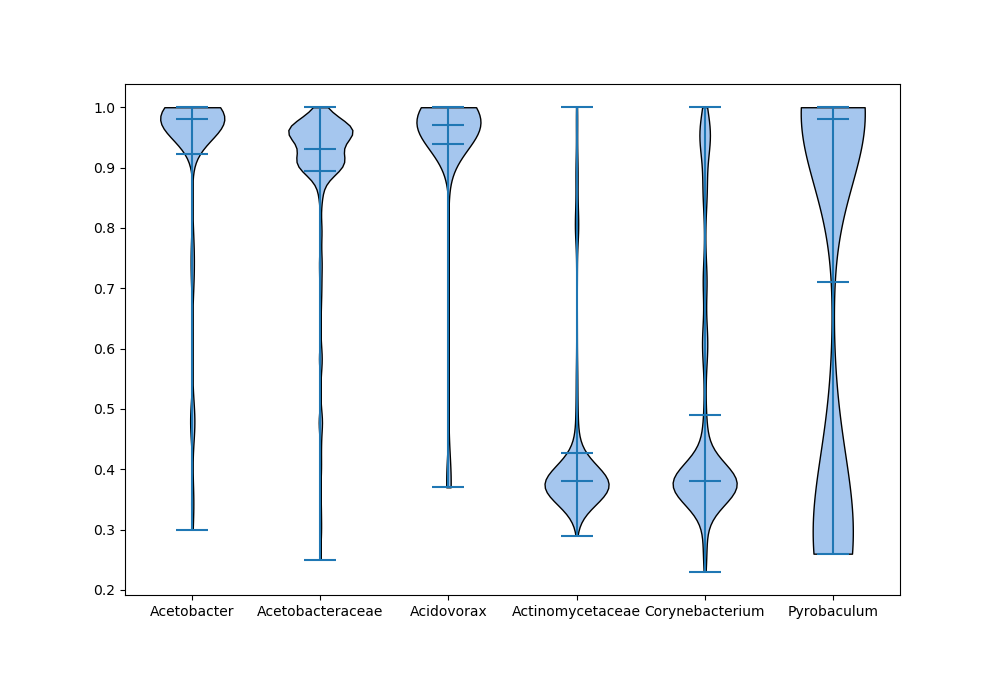


Figure D Identity distribution diagrams for six datasets of LTL_datasets

In this work, following evaluation scenarios have been taken into consideration based on the approaches used to assess the method's performance, as well as the diversity of the marker-based metagenomics data:

1. Employing the PC-mer method on two independent datasets, each with a different sequencing technology (AMP technology and SG technology).

2. Examining datasets with four different evolutionary levels of categorization: Class, Order, Family, and Genus.

3. Performance comparison of various classification methods (i.e. CNN, RDP, DBN) in terms of four metrics: accuracy, F1-score, precision, and recall, employing eight machine learning-based classifiers.

4. Evaluating training times of classifiers.

5. Evaluating various feature extraction methods for varying k-mer sizes by repeating all aforementioned tests for k-mer sizes of 3 to 12.

# Investigating the impact of size k in the PC-mer method

Table B Investigating the impact of size k in the PC-mer method generating input vectors for an LR classifier

| *k-mer* | *Metrics* | *DataSets (LR)* | | | | | | | |
| --- | --- | --- | --- | --- | --- | --- | --- | --- | --- |
|  |  | *AMP* | | | | *SG* | | | |
|  |  | *Class*  (%) | *Order*  (%) | *Family*  (%) | *Genus*  (%) | *Class*  (%) | *Order*  (%) | *Family*  (%) | *Genus*  (%) |
| 3 | *Accuracy* | 81.28 | 45.48 | 36.89 | 19.29 | 59.47 | 26.50 | 263 | 6.49 |
|  | *F1* | 79.41 | 43.19 | 32.01 | 17.42 | 57.37 | 22.10 | 13.06 | 5.10 |
|  | *Precision* | 79.60 | 44.18 | 31.58 | 17.21 | 57.46 | 22.66 | 13.97 | 5.29 |
|  | *Recall* | 81.28 | 45.48 | 36.89 | 19.29 | 59.47 | 26.50 | 263 | 6.49 |
| 4 | *Accuracy* | 86.45 | 62.18 | 54.41 | 409 | 63.61 | 33.63 | 26.47 | 180 |
|  | *F1* | 85.89 | 61.53 | 52.51 | 39.13 | 61.58 | 339 | 21.11 | 9.48 |
|  | *Precision* | 85.85 | 62.04 | 52.25 | 39.21 | 62.70 | 375 | 21.47 | 9.33 |
|  | *Recall* | 86.45 | 62.18 | 54.41 | 409 | 63.61 | 33.63 | 26.47 | 180 |
| 5 | *Accuracy* | 91.80 | 82.6 | 74.09 | 64.37 | 726 | 46.30 | 39.30 | 21.12 |
|  | *F1* | 91.69 | 80.5 | 73.78 | 64.08 | 69.29 | 44.40 | 36.20 | 213 |
|  | *Precision* | 91.66 | 82.4 | 73.86 | 64.43 | 69.47 | 44.48 | 36.12 | 19.99 |
|  | *Recall* | 91.80 | 82.6 | 74.09 | 64.37 | 726 | 46.30 | 39.30 | 21.12 |
| 6 | *Accuracy* | 96.95 | 92.10 | 89.15 | 82.63 | 83.17 | 63.66 | 58.46 | 39.14 |
|  | *F1* | 96.94 | 92.08 | 89.11 | 82.57 | 82.90 | 62.81 | 57.40 | 38.52 |
|  | *Precision* | 96.95 | 92.14 | 89.26 | 82.95 | 82.88 | 62.92 | 57.29 | 38.64 |
|  | *Recall* | 96.95 | 92.10 | 89.15 | 82.63 | 83.17 | 63.66 | 58.46 | 39.14 |
| 7 | *Accuracy* | 98.91 | 97.21 | 96.53 | 93.09 | 968 | 819 | 78.28 | 59.61 |
|  | *F1* | 98.91 | 97.21 | 96.52 | 93.08 | 963 | 79.92 | 78.11 | 59.48 |
|  | *Precision* | 98.91 | 97.23 | 96.58 | 93.26 | 961 | 79.99 | 78.29 | 608 |
|  | *Recall* | 98.91 | 97.21 | 96.53 | 93.09 | 968 | 819 | 78.28 | 59.61 |
| 8 | *Accuracy* | 99.80 | 99.45 | 99.39 | 96.95 | 96.49 | 958 | 946 | 76.59 |
|  | *F1* | 99.80 | 99.45 | 99.39 | 96.96 | 96.49 | 952 | 944 | 76.64 |
|  | *Precision* | 99.80 | 99.45 | 99.40 | 97.04 | 96.49 | 956 | 960 | 77.24 |
|  | *Recall* | 99.80 | 99.45 | 99.39 | 96.95 | 96.49 | 958 | 946 | 76.59 |
| 9 | *Accuracy* | 99.95 | 99.89 | 99.94 | 98.29 | 98.66 | 95.53 | 96.86 | 88.39 |
|  | *F1* | 99.95 | 99.89 | 99.94 | 98.29 | 98.66 | 95.52 | 96.85 | 88.39 |
|  | *Precision* | 99.95 | 99.89 | 99.94 | 98.31 | 98.66 | 95.54 | 96.90 | 88.67 |
|  | *Recall* | 99.95 | 99.89 | 99.94 | 98.29 | 98.67 | 95.53 | 96.86 | 88.39 |
| 10 | *Accuracy* | 99.99 | 99.98 | 99.99 | 98.52 | 99.69 | 98.69 | 99.31 | 94.22 |
|  | *F1* | 99.99 | 99.97 | 100 | 98.52 | 99.69 | 98.68 | 99.31 | 94.20 |
|  | *Precision* | 99.99 | 99.98 | 100 | 98.54 | 99.69 | 98.69 | 99.32 | 94.35 |
|  | *Recall* | 99.99 | 99.98 | 100 | 98.52 | 99.69 | 98.69 | 99.31 | 94.22 |
| 11 | *Accuracy* | 99.99 | 99.99 | 100 | 98.61 | 99.94 | 99.42 | 99.72 | 96.61 |
|  | *F1* | 99.99 | 99.99 | 100 | 98.60 | 99.94 | 99.41 | 99.72 | 96.58 |
|  | *Precision* | 99.99 | 99.99 | 100 | 98.62 | 99.94 | 99.41 | 99.73 | 96.69 |
|  | *Recall* | 99.99 | 99.99 | 100 | 98.61 | 99.94 | 99.42 | 99.72 | 96.61 |
| 12 | *Accuracy* | 100 | 100 | 100 | 98.64 | 100 | 99.74 | 99.87 | 97.51 |
|  | *F1* | 100 | 100 | 100 | 98.63 | 100 | 99.74 | 99.86 | 97.49 |
|  | *Precision* | 100 | 100 | 100 | 98.66 | 100 | 99.74 | 99.87 | 97.57 |
|  | *Recall* | 100 | 100 | 100 | 98.64 | 100 | 99.74 | 99.87 | 97.51 |

Table C Investigating the impact of size k in the PC-mer method generating input vectors for a DT classifier

| *k-mer* | *Metrics* | *DataSets(DT)* | | | | | | | |
| --- | --- | --- | --- | --- | --- | --- | --- | --- | --- |
|  |  | *AMP* | | | | *SG* | | | |
|  |  | *Class*  (%) | *Order*  (%) | *Family*  (%) | *Genus*  (%) | *Class*  (%) | *Order*  (%) | *Family*  (%) | *Genus*  (%) |
| 3 | *Accuracy* | 70.47 | 26.75 | 18.51 | 5.77 | 50.97 | 14.81 | 8.90 | 2.32 |
|  | *F1* | 70.71 | 26.84 | 18.63 | 5.74 | 51.13 | 14.84 | 8.89 | 2.29 |
|  | *Precision* | 70.97 | 26.99 | 18.82 | 5.82 | 51.30 | 14.92 | 8.92 | 2.32 |
|  | *Recall* | 70.47 | 26.75 | 18.51 | 5.77 | 50.97 | 14.81 | 8.90 | 2.32 |
| 4 | *Accuracy* | 74.82 | 34.86 | 25.52 | 9.58 | 53.27 | 53.23 | 12.14 | 3.06 |
|  | *F1* | 74.95 | 34.90 | 25.69 | 9.60 | 53.42 | 53.37 | 12.13 | 03.06 |
|  | *Precision* | 75.10 | 35.02 | 25.99 | 9.81 | 53.59 | 53.534 | 12.15 | 03.11 |
|  | *Recall* | 74.82 | 34.86 | 25.52 | 9.58 | 53.27 | 53.23 | 12.14 | 03.06 |
| 5 | *Accuracy* | 79.29 | 44.70 | 34.43 | 14.98 | 58.57 | 58.85 | 16.03 | 04.56 |
|  | *F1* | 79.36 | 44.72 | 34.51 | 14.94 | 58.66 | 58.94 | 16.06 | 04.53 |
|  | *Precision* | 79.45 | 44.85 | 34.73 | 15.18 | 58.77 | 59.04 | 16.16 | 04.59 |
|  | *Recall* | 79.29 | 44.70 | 34.43 | 14.98 | 58.57 | 58.85 | 16.03 | 04.56 |
| 6 | *Accuracy* | 85.04 | 56.79 | 44.86 | 24.02 | 66.63 | 63.65 | 22.42 | 06.69 |
|  | *F1* | 85.08 | 56.77 | 44.93 | 23.96 | 66.73 | 63.81 | 22.43 | 06.67 |
|  | *Precision* | 85.15 | 56.86 | 45.16 | 24.33 | 66.84 | 63.82 | 22.52 | 06.77 |
|  | *Recall* | 85.04 | 56.79 | 44.86 | 24.02 | 66.63 | 63.65 | 22.42 | 06.69 |
| 7 | *Accuracy* | 91.18 | 71.77 | 60.48 | 39.77 | 75.05 | 75.17 | 34.08 | 11.71 |
|  | *F1* | 91.20 | 71.80 | 60.44 | 39.72 | 75.12 | 75.21 | 34.08 | 11.60 |
|  | *Precision* | 91.24 | 71.99 | 60.66 | 40.19 | 75.20 | 75.27 | 34.21 | 11.70 |
|  | *Recall* | 91.18 | 71.77 | 60.48 | 39.77 | 75.05 | 75.17 | 34.08 | 11.71 |
| 8 | *Accuracy* | 96.94 | 84.84 | 76.31 | 61.94 | 90.11 | 90.06 | 56.43 | 29.26 |
|  | *F1* | 96.94 | 84.85 | 76.27 | 61.83 | 90.13 | 90.08 | 56.37 | 29.11 |
|  | *Precision* | 96.94 | 84.98 | 76.48 | 62.45 | 90.16 | 90.12 | 56.53 | 29.46 |
|  | *Recall* | 96.94 | 84.84 | 76.31 | 61.94 | 90.11 | 90.06 | 56.43 | 29.26 |
| 9 | *Accuracy* | 98.61 | 89.78 | 87.97 | 76.41 | 97.42 | 97.26 | 75.59 | 51.37 |
|  | *F1* | 98.61 | 89.77 | 87.96 | 76.38 | 97.42 | 97.26 | 75.55 | 51.23 |
|  | *Precision* | 98.61 | 89.87 | 88.14 | 76.90 | 97.42 | 97.27 | 75.74 | 51.79 |
|  | *Recall* | 98.61 | 89.78 | 87.97 | 76.41 | 97.42 | 97.26 | 75.59 | 51.37 |
| 10 | *Accuracy* | 99.22 | 95.83 | 93.64 | 85.69 | 98.67 | 98.64 | 85.36 | 68.64 |
|  | *F1* | 99.22 | 95.83 | 93.64 | 85.69 | 98.68 | 98.64 | 85.34 | 68.54 |
|  | *Precision* | 99.22 | 95.89 | 93.75 | 86.09 | 98.68 | 98.64 | 85.50 | 69.05 |
|  | *Recall* | 99.22 | 95.83 | 93.64 | 85.69 | 98.67 | 98.64 | 85.36 | 68.64 |
| 11 | *Accuracy* | 98.79 | 96.49 | 93.33 | 90.82 | 99.09 | 99.06 | 88.58 | 77.19 |
|  | *F1* | 98.79 | 96.49 | 93.33 | 90.80 | 99.09 | 99.06 | 88.54 | 77.11 |
|  | *Precision* | 98.79 | 96.54 | 93.48 | 91.10 | 99.09 | 99.06 | 88.68 | 77.53 |
|  | *Recall* | 98.79 | 96.49 | 93.33 | 90.82 | 99.09 | 99.06 | 88.58 | 77.19 |
| 12 | *Accuracy* | 98.98 | 98.07 | 95.35 | 93.48 | 99.31 | 99.34 | 91.91 | 82.18 |
|  | *F1* | 98.98 | 98.07 | 95.34 | 93.47 | 99.31 | 99.34 | 91.90 | 82.13 |
|  | *Precision* | 98.98 | 98.10 | 95.43 | 93.65 | 99.31 | 99.34 | 92.02 | 82.53 |
|  | *Recall* | 98.98 | 98.07 | 95.35 | 93.48 | 99.31 | 99.34 | 91.91 | 82.18 |

Table D Investigating the impact of size k in the PC-mer method generating input vectors for a GNB classifier

| *k-mer* | *Metrics* | *DataSets(GNB)* | | | | | | | |
| --- | --- | --- | --- | --- | --- | --- | --- | --- | --- |
|  |  | *AMP* | | | | *SG* | | | |
|  |  | *Class*  (%) | *Order*  (%) | *Family*  (%) | *Genus*  (%) | *Class*  (%) | *Order*  (%) | *Family*  (%) | *Genus*  (%) |
| 3 | *Accuracy* | 78.27 | 38.39 | 29.04 | 14.11 | 42.60 | 9.64 | 6.52 | 4.43 |
|  | *F1* | 78.57 | 38.47 | 28.41 | 12.91 | 46.43 | 10.23 | 6.03 | 3.61 |
|  | *Precision* | 78.92 | 39.68 | 30.54 | 14.62 | 55.60 | 21.23 | 14.56 | 3.88 |
|  | *Recall* | 78.27 | 38.39 | 29.04 | 14.11 | 42.60 | 9.64 | 6.52 | 4.43 |
| 4 | *Accuracy* | 82.91 | 53.28 | 44.27 | 29.97 | 47.17 | 16.1 | 11.27 | 6.94 |
|  | *F1* | 83.21 | 53.40 | 44.35 | 29.26 | 50.98 | 17.25 | 11.43 | 6.17 |
|  | *Precision* | 83.64 | 54.67 | 46.14 | 30.68 | 58.60 | 27.39 | 19.33 | 6.68 |
|  | *Recall* | 82.91 | 53.28 | 44.27 | 29.97 | 47.17 | 16.1 | 11.27 | 6.94 |
| 5 | *Accuracy* | 86.80 | 70.10 | 64.06 | 54.06 | 54.18 | 24.46 | 18.55 | 11.83 |
|  | *F1* | 87.18 | 70.29 | 64.09 | 53.67 | 56.95 | 25.68 | 19.67 | 11.18 |
|  | *Precision* | 87.87 | 71.69 | 65.28 | 54.41 | 63.16 | 35.55 | 29.49 | 11.6 |
|  | *Recall* | 86.80 | 70.10 | 64.06 | 54.06 | 54.18 | 24.46 | 18.55 | 11.83 |
| 6 | *Accuracy* | 92.71 | 84.98 | 81.53 | 77.05 | 62.69 | 35.33 | 30.32 | 19.89 |
|  | *F1* | 92.90 | 85.08 | 81.59 | 76.92 | 65.21 | 36.89 | 32.05 | 19.51 |
|  | *Precision* | 93.31 | 85.63 | 82.20 | 77.44 | 70.39 | 44.6 | 40.01 | 20.24 |
|  | *Recall* | 92.71 | 84.98 | 81.53 | 77.05 | 62.69 | 35.33 | 30.32 | 19.89 |
| 7 | *Accuracy* | 97.02 | 93.77 | 92.87 | 90.53 | 70.73 | 47.92 | 45.55 | 31.88 |
|  | *F1* | 97.06 | 93.79 | 92.90 | 90.52 | 72.37 | 49.58 | 47.12 | 31.96 |
|  | *Precision* | 97.16 | 93.90 | 93.08 | 90.81 | 75.90 | 56.37 | 52.92 | 33.3 |
|  | *Recall* | 97.02 | 93.77 | 92.87 | 90.53 | 70.73 | 47.92 | 45.55 | 31.88 |
| 8 | *Accuracy* | 98.47 | 96.66 | 97.30 | 95.54 | 76.96 | 62.04 | 61.52 | 45.12 |
|  | *F1* | 98.48 | 96.66 | 97.31 | 95.54 | 78.00 | 63.18 | 62.62 | 45.46 |
|  | *Precision* | 98.50 | 96.71 | 97.38 | 95.68 | 80.36 | 67.56 | 65.97 | 46.88 |
|  | *Recall* | 98.47 | 96.66 | 97.30 | 95.54 | 76.96 | 62.04 | 61.52 | 45.12 |
| 9 | *Accuracy* | 99.20 | 97.70 | 98.87 | 97.39 | 84.03 | 72.68 | 72.79 | 57.92 |
|  | *F1* | 99.20 | 97.70 | 98.87 | 97.39 | 85.12 | 73.75 | 73.68 | 58.26 |
|  | *Precision* | 99.20 | 97.74 | 98.89 | 97.47 | 87.69 | 77.19 | 76.85 | 59.93 |
|  | *Recall* | 99.20 | 97.70 | 98.87 | 97.39 | 84.03 | 72.68 | 72.79 | 57.92 |
| 10 | *Accuracy* | 99.55 | 98.61 | 99.50 | 97.97 | 87.54 | 82.82 | 90.51 | 73.32 |
|  | *F1* | 99.55 | 98.61 | 99.50 | 97.97 | 88.07 | 83.46 | 90.62 | 73.27 |
|  | *Precision* | 99.55 | 98.63 | 99.50 | 98.04 | 89.52 | 87 | 91.16 | 74.2 |
|  | *Recall* | 99.55 | 98.61 | 99.50 | 97.97 | 87.54 | 82.82 | 90.51 | 73.32 |
| 11 | *Accuracy* | 99.75 | 99.36 | 99.77 | 97.89 | 81.49 | 82.8 | 93.33 | 79.92 |
|  | *F1* | 99.75 | 99.36 | 99.77 | 97.89 | 82.71 | 83.47 | 93.55 | 79.32 |
|  | *Precision* | 99.75 | 99.37 | 99.77 | 97.94 | 86.52 | 87.87 | 94.27 | 80.06 |
|  | *Recall* | 99.75 | 99.36 | 99.77 | 97.89 | 81.49 | 82.8 | 93.33 | 79.92 |
| 12 | *Accuracy* | 99.88 | 99.01 | 98.28 | 90.44 | 76.36 | 80.79 | 93.08 | 80.21 |
|  | *F1* | 99.88 | 98.99 | 98.25 | 90.35 | 75.49 | 80.94 | 93.4 | 79.69 |
|  | *Precision* | 99.88 | 99.05 | 98.43 | 91.27 | 82.34 | 85.89 | 94.45 | 80.49 |
|  | *Recall* | 99.88 | 99.0142 | 98.28 | 90.44 | 76.36 | 80.79 | 93.08 | 80.21 |

Table E Investigating the impact of size k in the PC-mer method generating input vectors for an LDA classifier

| *k-mer* | *Metrics* | *DataSets(LDA)* | | | | | | | |
| --- | --- | --- | --- | --- | --- | --- | --- | --- | --- |
|  |  | *AMP* | | | | *SG* | | | |
|  |  | *Class*  (%) | *Order*  (%) | *Family*  (%) | *Genus*  (%) | *Class*  (%) | *Order*  (%) | *Family*  (%) | *Genus*  (%) |
| 3 | *Accuracy* | 81.45 | 45.99 | 37.42 | 19.33 | 59.06 | 25.93 | 21.05 | 6.24 |
|  | *F1* | 79.69 | 43.85 | 32.76 | 17.66 | 57.04 | 21.72 | 13.37 | 5 |
|  | *Precision* | 79.87 | 45.04 | 32.76 | 17.4 | 61.84 | 22.82 | 15.72 | 5.09 |
|  | *Recall* | 81.45 | 45.99 | 37.42 | 19.33 | 59.06 | 25.93 | 21.05 | 6.24 |
| 4 | *Accuracy* | 86.73 | 62.88 | 54.86 | 39.93 | 63.98 | 34.33 | 26.5 | 10.03 |
|  | *F1* | 86.29 | 62.23 | 53.26 | 38.91 | 62.36 | 31 | 21.63 | 9.14 |
|  | *Precision* | 86.22 | 62.59 | 52.9 | 38.71 | 63.03 | 31.06 | 22.6 | 9.29 |
|  | *Recall* | 86.73 | 62.88 | 54.86 | 39.93 | 63.98 | 34.33 | 26.5 | 10.03 |
| 5 | *Accuracy* | 91.87 | 80.5 | 74.65 | 64.61 | 70.71 | 45.28 | 39.29 | 19.17 |
|  | *F1* | 91.77 | 80.3 | 74.45 | 64.4 | 69.86 | 43.57 | 36.61 | 18.43 |
|  | *Precision* | 91.75 | 80.45 | 74.56 | 64.73 | 69.98 | 43.64 | 36.11 | 18.38 |
|  | *Recall* | 91.87 | 80.5 | 74.65 | 64.61 | 70.71 | 45.28 | 39.29 | 19.17 |
| 6 | *Accuracy* | 96.97 | 92.22 | 89.51 | 85 | 83.14 | 62.92 | 57.82 | 36.85 |
|  | *F1* | 96.95 | 92.2 | 89.5 | 84.91 | 82.89 | 62.11 | 56.61 | 36.21 |
|  | *Precision* | 96.95 | 92.26 | 89.65 | 85.17 | 82.89 | 62.39 | 56.39 | 36.36 |
|  | *Recall* | 96.97 | 92.22 | 89.51 | 85 | 83.14 | 62.92 | 57.82 | 36.85 |
| 7 | *Accuracy* | 99.19 | 97.79 | 97.27 | 94.9 | 90.37 | 80.27 | 79.14 | 59.59 |
|  | *F1* | 99.19 | 97.79 | 97.27 | 94.89 | 90.28 | 80.11 | 78.86 | 59.19 |
|  | *Precision* | 99.19 | 97.81 | 97.31 | 94.99 | 90.24 | 80.26 | 78.92 | 59.59 |
|  | *Recall* | 99.19 | 97.79 | 97.27 | 94.9 | 90.37 | 80.27 | 79.14 | 59.59 |
| 8 | *Accuracy* | 99.84 | 99.48 | 99.42 | 97.4 | 96.92 | 91.04 | 92.32 | 78.29 |
|  | *F1* | 99.84 | 99.48 | 99.42 | 97.4 | 96.92 | 91.04 | 92.26 | 78.21 |
|  | *Precision* | 99.84 | 99.48 | 99.44 | 97.47 | 96.92 | 91.1 | 92.39 | 78.78 |
|  | *Recall* | 99.84 | 99.48 | 99.42 | 97.4 | 96.92 | 91.04 | 92.32 | 78.29 |
| 9 | *Accuracy* | 99.97 | 99.88 | 99.92 | 98.25 | 99.01 | 96.29 | 97.45 | 88.21 |
|  | *F1* | 99.97 | 99.88 | 99.92 | 98.25 | 99.01 | 96.24 | 97.45 | 88.2 |
|  | *Precision* | 99.97 | 99.88 | 99.92 | 98.31 | 99.01 | 96.26 | 97.49 | 88.51 |
|  | *Recall* | 99.97 | 99.88 | 99.92 | 98.25 | 99.01 | 96.29 | 97.45 | 88.21 |
| 10 | *Accuracy* | 100 | 99.97 | 100 | 98.47 | 99.86 | 98.3 | 99.15 | 92.92 |
|  | *F1* | 100 | 99.97 | 100 | 98.45 | 99.86 | 98.29 | 99.15 | 92.92 |
|  | *Precision* | 100 | 99.97 | 100 | 98.53 | 99.86 | 98.31 | 99.16 | 93.26 |
|  | *Recall* | 100 | 99.97 | 100 | 98.47 | 99.86 | 98.3 | 99.15 | 92.92 |
| 11 | *Accuracy* | 100 | 100 | 100 | 98.54 | 100 | 99.08 | 99.33 | 94.73 |
|  | *F1* | 100 | 100 | 100 | 98.53 | 100 | 99.08 | 99.33 | 94.72 |
|  | *Precision* | 100 | 100 | 100 | 98.56 | 100 | 99.09 | 99.34 | 94.94 |
|  | *Recall* | 100 | 100 | 100 | 98.54 | 100 | 99.08 | 99.33 | 94.73 |
| 12 | *Accuracy* | 100 | 100 | 100 | 99.11 | 100 | 99.31 | 99.54 | 96.88 |
|  | *F1* | 100 | 100 | 100 | 99.11 | 100 | 99.44 | 99.53 | 96.55 |
|  | *Precision* | 100 | 100 | 100 | 99.21 | 100 | 99.44 | 99.65 | 96.98 |
|  | *Recall* | 100 | 100 | 100 | 99.11 | 100 | 99.31 | 99.54 | 96.88 |

Table F Investigating the impact of size k in the PC-mer method generating input vectors for an MLP classifier

| *k-mer* | *Metrics* | *DataSets(MLP)* | | | | | | | |
| --- | --- | --- | --- | --- | --- | --- | --- | --- | --- |
|  |  | *AMP* | | | | *SG* | | | |
|  |  | *Class*  (%) | *Order*  (%) | *Family*  (%) | *Genus*  (%) | *Class*  (%) | *Order*  (%) | *Family*  (%) | *Genus*  (%) |
| 3 | *Accuracy* | 80.35 | 44.76 | 37.02 | 18.4 | 52.33 | 24.05 | 19.77 | 5.45 |
|  | *F1* | 78.66 | 42.6 | 31.99 | 16.45 | 42.66 | 18.58 | 11.92 | 3.88 |
|  | *Precision* | 79.21 | 44.59 | 32.2 | 17.46 | 56.36 | 24.93 | 13.87 | 4.5 |
|  | *Recall* | 80.35 | 44.76 | 37.02 | 18.4 | 52.33 | 24.05 | 19.77 | 5.45 |
| 4 | *Accuracy* | 85.57 | 62.04 | 54.01 | 37.49 | 59.41 | 32.75 | 25.2 | 8.08 |
|  | *F1* | 85.13 | 61.32 | 51.89 | 36.38 | 53.31 | 29.02 | 18.96 | 6.25 |
|  | *Precision* | 85.68 | 62.66 | 52.44 | 38.35 | 64.54 | 30.81 | 19.66 | 6.74 |
|  | *Recall* | 85.57 | 62.04 | 54.01 | 37.49 | 59.41 | 32.75 | 25.2 | 8.08 |
| 5 | *Accuracy* | 93.63 | 80.31 | 66.76 | 54.48 | 73.3 | 44.65 | 34.51 | 13.86 |
|  | *F1* | 93.59 | 80.07 | 65.69 | 53.85 | 72.1 | 42.5 | 28.9 | 12.24 |
|  | *Precision* | 93.66 | 80.73 | 66.21 | 55.5 | 74.57 | 43.41 | 29.02 | 13.16 |
|  | *Recall* | 93.63 | 80.31 | 66.76 | 54.48 | 73.3 | 44.65 | 34.51 | 13.86 |
| 6 | *Accuracy* | 97.82 | 91.85 | 85.33 | 66.19 | 88.66 | 62.61 | 49.25 | 22.51 |
|  | *F1* | 97.82 | 91.84 | 85.26 | 65.85 | 88.51 | 61.2 | 45.63 | 20.97 |
|  | *Precision* | 97.82 | 92.01 | 85.78 | 67.33 | 88.88 | 62.85 | 46.73 | 22.72 |
|  | *Recall* | 97.82 | 91.85 | 85.33 | 66.19 | 88.66 | 62.61 | 49.25 | 22.51 |
| 7 | *Accuracy* | 99.32 | 97 | 92.89 | 81.08 | 94.35 | 75.1 | 69.38 | 30.64 |
|  | *F1* | 99.32 | 97 | 92.88 | 80.98 | 94.37 | 73.84 | 68.35 | 28.9 |
|  | *Precision* | 99.32 | 97.04 | 93.06 | 81.79 | 94.41 | 74.57 | 68.88 | 30.01 |
|  | *Recall* | 99.32 | 97 | 92.89 | 81.08 | 94.35 | 75.1 | 69.38 | 30.64 |
| 8 | *Accuracy* | 99.79 | 99.43 | 98.68 | 85.17 | 97.63 | 88.6 | 84.17 | 44.34 |
|  | *F1* | 99.79 | 99.43 | 98.67 | 85.05 | 97.64 | 88.58 | 84.15 | 43.01 |
|  | *Precision* | 99.79 | 99.43 | 98.7 | 85.62 | 97.64 | 88.71 | 84.47 | 44.21 |
|  | *Recall* | 99.79 | 99.43 | 98.68 | 85.17 | 97.63 | 88.6 | 84.17 | 44.34 |
| 9 | *Accuracy* | 99.95 | 99.84 | 99.71 | 96.16 | 98.88 | 95.09 | 93.24 | 63.39 |
|  | *F1* | 99.95 | 99.84 | 99.71 | 96.16 | 98.88 | 95.07 | 93.2 | 63.13 |
|  | *Precision* | 99.95 | 99.84 | 99.71 | 96.26 | 98.88 | 95.1 | 93.32 | 63.97 |
|  | *Recall* | 99.95 | 99.84 | 99.71 | 96.16 | 98.88 | 95.09 | 93.24 | 63.39 |
| 10 | *Accuracy* | 99.99 | 99.96 | 99.96 | 98.22 | 99.67 | 98.5 | 97.92 | 81.14 |
|  | *F1* | 99.99 | 99.96 | 99.96 | 98.21 | 99.67 | 98.49 | 97.91 | 81.09 |
|  | *Precision* | 99.99 | 99.96 | 99.96 | 98.24 | 99.67 | 98.5 | 97.96 | 81.5 |
|  | *Recall* | 99.99 | 99.96 | 99.96 | 98.22 | 99.67 | 98.5 | 97.92 | 81.14 |
| 11 | *Accuracy* | 100 | 99.98 | 99.99 | 98.44 | 99.92 | 99.22 | 99.27 | 90.72 |
|  | *F1* | 100 | 99.98 | 99.99 | 98.44 | 99.92 | 99.21 | 99.27 | 90.66 |
|  | *Precision* | 100 | 99.98 | 99.99 | 98.45 | 99.92 | 99.22 | 99.29 | 90.88 |
|  | *Recall* | 100 | 99.98 | 99.99 | 98.44 | 99.92 | 99.22 | 99.27 | 90.72 |
| 12 | *Accuracy* | 100 | 100 | 99.99 | 98.6 | 99.97 | 99.68 | 99.69 | 94.95 |
|  | *F1* | 100 | 100 | 99.99 | 98.59 | 99.97 | 99.68 | 99.69 | 94.91 |
|  | *Precision* | 100 | 100 | 99.99 | 98.61 | 99.97 | 99.68 | 99.7 | 95.05 |
|  | *Recall* | 100 | 100 | 99.99 | 98.6 | 99.97 | 99.68 | 99.69 | 94.95 |

Table G Investigating the impact of size k in the PC-mer method generating input vectors for an SVC classifier

| *k-mer* | *Metrics* | *DataSets(SVC)* | | | | | | | |
| --- | --- | --- | --- | --- | --- | --- | --- | --- | --- |
|  |  | *AMP* | | | | *SG* | | | |
|  |  | *Class*  (%) | *Order*  (%) | *Family*  (%) | *Genus*  (%) | *Class*  (%) | *Order*  (%) | *Family*  (%) | *Genus*  (%) |
| 3 | *Accuracy* | 74.53 | 3.73 | 8.22 | 1.29 | 46.16 | 2.91 | 12.54 | 1.03 |
|  | *F1* | 71.13 | 3.2 | 5.91 | 0.54 | 29.16 | 0.17 | 9.93 | 0.03 |
|  | *Precision* | 75.68 | 4.49 | 15.39 | 2.18 | 21.31 | 0.09 | 13.18 | 0.02 |
|  | *Recall* | 74.53 | 3.73 | 8.22 | 1.29 | 46.16 | 2.91 | 12.54 | 1.03 |
| 4 | *Accuracy* | 72.54 | 36.15 | 21.79 | 22.97 | 51.01 | 19.91 | 1.14 | 0.89 |
|  | *F1* | 73.77 | 26.61 | 19.47 | 20.75 | 40.22 | 9.33 | 0.03 | 0.02 |
|  | *Precision* | 85.82 | 49.26 | 36.37 | 35.87 | 60.54 | 7.05 | 0.02 | 0.08 |
|  | *Recall* | 72.54 | 36.15 | 21.79 | 22.97 | 51.01 | 19.91 | 1.14 | 0.89 |
| 5 | *Accuracy* | 87.14 | 67.86 | 50.72 | 22.25 | 52.89 | 7.9 | 30.11 | 1.88 |
|  | *F1* | 86.85 | 67.14 | 46.48 | 26.7 | 52.87 | 4.3 | 25.21 | 0.13 |
|  | *Precision* | 89.51 | 77.52 | 68.07 | 59.7 | 70.11 | 33.85 | 30.02 | 0.07 |
|  | *Recall* | 87.14 | 67.86 | 50.72 | 22.25 | 52.89 | 7.9 | 30.11 | 1.88 |
| 6 | *Accuracy* | 95.97 | 83.93 | 82.5 | 70.5 | 70.67 | 35.32 | 39.79 | 18.18 |
|  | *F1* | 95.94 | 82.75 | 82.34 | 71.91 | 67.68 | 34.5 | 39.86 | 18.25 |
|  | *Precision* | 96.1 | 86.49 | 85.27 | 81 | 78.89 | 62.86 | 57.46 | 33.29 |
|  | *Recall* | 95.97 | 83.93 | 82.5 | 70.5 | 70.67 | 35.32 | 39.79 | 18.18 |
| 7 | *Accuracy* | 99.07 | 95.33 | 95.08 | 88.61 | 89.02 | 67.59 | 70.46 | 42.58 |
|  | *F1* | 99.06 | 95.31 | 95.13 | 88.85 | 88.26 | 67.45 | 70.27 | 43.71 |
|  | *Precision* | 99.07 | 95.58 | 95.29 | 89.88 | 89.25 | 73.46 | 73.48 | 56.4 |
|  | *Recall* | 99.07 | 95.33 | 95.08 | 88.61 | 89.02 | 67.59 | 70.46 | 42.58 |
| 8 | *Accuracy* | 99.75 | 98.29 | 97.93 | 96.47 | 95.61 | 85.8 | 85.16 | 63.59 |
|  | *F1* | 99.75 | 98.29 | 97.94 | 96.35 | 95.54 | 85.78 | 85.17 | 63.6 |
|  | *Precision* | 99.75 | 98.31 | 98 | 96.37 | 95.68 | 86.38 | 85.68 | 64.44 |
|  | *Recall* | 99.75 | 98.29 | 97.93 | 96.47 | 95.61 | 85.8 | 85.16 | 63.59 |
| 9 | *Accuracy* | 99.97 | 99.58 | 99.9 | 97.68 | 98.55 | 93.1 | 93.84 | 81.9 |
|  | *F1* | 99.97 | 99.58 | 99.9 | 97.62 | 98.55 | 93.09 | 93.82 | 81.69 |
|  | *Precision* | 99.96 | 99.58 | 99.9 | 97.58 | 98.56 | 93.17 | 93.9 | 82.12 |
|  | *Recall* | 99.97 | 99.58 | 99.9 | 97.68 | 98.55 | 93.1 | 93.84 | 81.9 |
| 10 | *Accuracy* | 100 | 99.97 | 99.97 | 98.29 | 99.69 | 96.57 | 98.38 | 92.18 |
|  | *F1* | 100 | 99.97 | 99.97 | 98.26 | 99.69 | 96.54 | 98.36 | 92.02 |
|  | *Precision* | 100 | 99.97 | 99.97 | 98.25 | 99.69 | 96.56 | 98.36 | 92.29 |
|  | *Recall* | 100 | 99.97 | 99.97 | 98.29 | 99.69 | 96.57 | 98.38 | 92.18 |
| 11 | *Accuracy* | 100 | 100 | 100 | 98.33 | 99.97 | 98.94 | 99.62 | 95.4 |
|  | *F1* | 100 | 100 | 100 | 98.31 | 99.97 | 98.93 | 99.61 | 95.35 |
|  | *Precision* | 100 | 100 | 100 | 98.31 | 99.97 | 98.94 | 99.62 | 95.49 |
|  | *Recall* | 100 | 100 | 100 | 98.33 | 99.97 | 98.94 | 99.62 | 95.4 |
| 12 | *Accuracy* | 100 | 100 | 100 | 98.47 | 100 | 99.58 | 99.79 | 97.03 |
|  | *F1* | 100 | 100 | 100 | 98.46 | 100 | 99.58 | 99.79 | 97.01 |
|  | *Precision* | 100 | 100 | 100 | 98.47 | 100 | 99.58 | 99.8 | 97.14 |
|  | *Recall* | 100 | 100 | 100 | 98.47 | 100 | 99.58 | 99.79 | 97.03 |

Table H Investigating the impact of size k in the PC-mer method generating input vectors for an NC-mean classifier

| *k-mer* | *Metrics* | *DataSets(NC-mean)* | | | | | | | |
| --- | --- | --- | --- | --- | --- | --- | --- | --- | --- |
|  |  | *AMP* | | | | *SG* | | | |
|  |  | *Class*  (%) | *Order*  (%) | *Family*  (%) | *Genus*  (%) | *Class*  (%) | *Order*  (%) | *Family*  (%) | *Genus*  (%) |
| 3 | *Accuracy* | 69.72 | 27.52 | 21.86 | 14.7 | 44.73 | 10.62 | 7.31 | 5.17 |
|  | *F1* | 73.14 | 29.1 | 22.68 | 13.27 | 48.82 | 11.06 | 6.49 | 4.06 |
|  | *Precision* | 79.33 | 42.26 | 32.38 | 14.44 | 57.22 | 22.66 | 16.49 | 4.58 |
|  | *Recall* | 69.72 | 27.52 | 21.86 | 14.7 | 44.73 | 10.62 | 7.31 | 5.17 |
| 4 | *Accuracy* | 79.31 | 46.99 | 40.24 | 31.24 | 50.2 | 17.71 | 12.67 | 8.5 |
|  | *F1* | 81.2 | 47.89 | 41.39 | 30.27 | 53.8 | 18.78 | 12.64 | 7.41 |
|  | *Precision* | 84.81 | 56.04 | 47.96 | 31.48 | 60.85 | 30.30 | 23.22 | 7.89 |
|  | *Recall* | 79.31 | 46.99 | 40.24 | 31.24 | 50.2 | 17.71 | 12.67 | 8.5 |
| 5 | *Accuracy* | 85.91 | 65.87 | 61.54 | 54.83 | 58.58 | 27.63 | 21.9 | 14.61 |
|  | *F1* | 86.98 | 66.56 | 62.03 | 54.32 | 61.18 | 29.3 | 23.01 | 13.66 |
|  | *Precision* | 89.21 | 71.68 | 65.17 | 55.11 | 66.49 | 40.17 | 33.23 | 14.42 |
|  | *Recall* | 85.91 | 65.87 | 61.54 | 54.83 | 58.58 | 27.63 | 21.9 | 14.61 |
| 6 | *Accuracy* | 91.91 | 82.08 | 80.17 | 77.74 | 70.7 | 41.39 | 36.82 | 25.6 |
|  | *F1* | 92.41 | 82.51 | 80.4 | 77.9 | 72.35 | 43.16 | 38.51 | 25.02 |
|  | *Precision* | 93.58 | 84.67 | 81.88 | 78.25 | 75.88 | 52.41 | 46.94 | 26.13 |
|  | *Recall* | 91.91 | 82.08 | 80.17 | 77.9 | 70.7 | 41.39 | 36.82 | 25.6 |
| 7 | *Accuracy* | 96.29 | 92.99 | 92.09 | 91 | 80.6 | 59.31 | 58.36 | 43.11 |
|  | *F1* | 96.41 | 93.07 | 92.18 | 90.97 | 81.66 | 60.73 | 59.48 | 43.02 |
|  | *Precision* | 96.73 | 93.45 | 92.64 | 91.26 | 83.99 | 66.99 | 63.97 | 44.42 |
|  | *Recall* | 96.29 | 92.99 | 92.09 | 91 | 80.6 | 59.31 | 58.36 | 43.11 |
| 8 | *Accuracy* | 98.15 | 96.69 | 97.08 | 95.91 | 89.04 | 77.04 | 78.72 | 62.46 |
|  | *F1* | 98.17 | 96.7 | 97.11 | 95.91 | 89.61 | 77.76 | 79.15 | 62.56 |
|  | *Precision* | 98.25 | 96.77 | 97.27 | 96.06 | 90.99 | 80.53 | 80.66 | 63.7 |
|  | *Recall* | 98.15 | 96.69 | 97.08 | 95.91 | 89.04 | 77.04 | 78.72 | 62.46 |
| 9 | *Accuracy* | 99.07 | 97.26 | 98.27 | 97.4 | 93.72 | 88.18 | 91.26 | 75.71 |
|  | *F1* | 99.07 | 97.25 | 98.29 | 97.39 | 93.98 | 88.5 | 91.37 | 75.84 |
|  | *Precision* | 99.09 | 97.35 | 98.4 | 97.51 | 94.66 | 89.64 | 91.74 | 76.67 |
|  | *Recall* | 99.07 | 97.26 | 98.27 | 97.4 | 93.72 | 88.18 | 91.26 | 75.71 |
| 10 | *Accuracy* | 99.33 | 97.03 | 98.71 | 97.98 | 96.49 | 94.11 | 96.55 | 83.52 |
|  | *F1* | 99.33 | 97.02 | 98.73 | 97.96 | 96.57 | 94.27 | 96.58 | 83.68 |
|  | *Precision* | 99.34 | 97.17 | 98.82 | 98.08 | 96.82 | 94.79 | 96.7 | 84.34 |
|  | *Recall* | 99.33 | 97.03 | 98.71 | 97.98 | 96.49 | 94.11 | 96.55 | 83.52 |
| 11 | *Accuracy* | 99.44 | 96.94 | 98.78 | 98.12 | 98 | 96.56 | 98.34 | 87.07 |
|  | *F1* | 99.44 | 96.93 | 98.79 | 98.1 | 98.03 | 96.67 | 98.34 | 87.23 |
|  | *Precision* | 99.44 | 97.12 | 98.89 | 98.22 | 98.1 | 97 | 98.39 | 87.82 |
|  | *Recall* | 99.44 | 96.94 | 98.78 | 98.12 | 98 | 96.56 | 98.34 | 87.07 |
| 12 | *Accuracy* | 99.37 | 96.63 | 98.79 | 98.21 | 98.6 | 97.4 | 98.89 | 89.09 |
|  | *F1* | 99.37 | 96.61 | 98.81 | 98.17 | 98.61 | 97.48 | 98.88 | 89.23 |
|  | *Precision* | 99.38 | 96.85 | 98.91 | 98.31 | 98.63 | 97.75 | 98.91 | 89.73 |
|  | *Recall* | 99.37 | 96.63 | 98.79 | 98.21 | 98.6 | 97.4 | 98.89 | 89.09 |

Table I Investigating the impact of size k in the PC-mer method generating input vectors for an NC-median classifier

| *k-mer* | *Metrics* | *DataSets(NC-median)* | | | | | | | |
| --- | --- | --- | --- | --- | --- | --- | --- | --- | --- |
|  |  | *AMP* | | | | *SG* | | | |
|  |  | *Class*  (%) | *Order*  (%) | *Family*  (%) | *Genus*  (%) | *Class*  (%) | *Order*  (%) | *Family*  (%) | *Genus*  (%) |
| 3 | *Accuracy* | 64.45 | 24.42 | 19.42 | 12.48 | 42.61 | 9.64 | 6.52 | 4.43 |
|  | *F1* | 68.64 | 25.87 | 20.46 | 11.3 | 46.43 | 10.24 | 6.03 | 3.61 |
|  | *Precision* | 76.45 | 38.67 | 30.3 | 12.03 | 55.61 | 21.23 | 14.56 | 3.88 |
|  | *Recall* | 64.45 | 24.42 | 19.42 | 12.48 | 42.61 | 9.64 | 6.52 | 4.43 |
| 4 | *Accuracy* | 75.71 | 42.69 | 36.63 | 26.48 | 47.17 | 16.1 | 11.27 | 6.94 |
|  | *F1* | 77.9 | 43.44 | 37.74 | 25.64 | 50.99 | 17.26 | 11.43 | 6.17 |
|  | *Precision* | 82.09 | 51.17 | 43.53 | 26.56 | 58.61 | 27.39 | 19.33 | 6.68 |
|  | *Recall* | 75.71 | 42.69 | 36.63 | 26.48 | 47.17 | 16.1 | 11.27 | 6.94 |
| 5 | *Accuracy* | 82.37 | 61.21 | 56.66 | 48.99 | 54.18 | 24.46 | 18.55 | 11.83 |
|  | *F1* | 83.97 | 61.73 | 57.27 | 48.57 | 56.96 | 25.68 | 19.67 | 11.18 |
|  | *Precision* | 87.23 | 67.39 | 60.75 | 49.35 | 63.17 | 35.55 | 29.49 | 11.6 |
|  | *Recall* | 82.37 | 61.21 | 56.66 | 48.99 | 54.18 | 24.46 | 18.55 | 11.83 |
| 6 | *Accuracy* | 89.8 | 78.32 | 74.98 | 72.14 | 62.7 | 35.33 | 30.32 | 19.89 |
|  | *F1* | 90.46 | 78.83 | 75.36 | 71.96 | 65.21 | 36.89 | 32.05 | 19.51 |
|  | *Precision* | 91.93 | 81.55 | 77.15 | 72.56 | 70.4 | 44.6 | 40.01 | 20.24 |
|  | *Recall* | 89.8 | 78.32 | 74.98 | 72.14 | 62.7 | 35.33 | 30.32 | 19.89 |
| 7 | *Accuracy* | 94.87 | 89.25 | 88.25 | 87.95 | 70.73 | 47.92 | 45.55 | 31.88 |
|  | *F1* | 95.09 | 89.46 | 88.39 | 87.9 | 72.38 | 49.58 | 47.12 | 31.96 |
|  | *Precision* | 95.66 | 90.48 | 89.2 | 88.28 | 75.91 | 56.37 | 52.92 | 33.3 |
|  | *Recall* | 94.87 | 89.25 | 88.25 | 87.95 | 70.73 | 47.92 | 45.55 | 31.88 |
| 8 | *Accuracy* | 96.18 | 93.72 | 94.31 | 92.84 | 76.96 | 62.04 | 61.52 | 45.12 |
|  | *F1* | 96.29 | 93.79 | 94.34 | 92.84 | 78 | 63.18 | 62.62 | 45.46 |
|  | *Precision* | 96.61 | 94.07 | 94.52 | 93.1 | 80.37 | 67.56 | 65.97 | 46.88 |
|  | *Recall* | 96.18 | 93.72 | 94.31 | 92.84 | 76.96 | 62.04 | 61.52 | 45.12 |
| 9 | *Accuracy* | 97.15 | 95.04 | 95.67 | 95.54 | 84.03 | 72.68 | 72.79 | 57.92 |
|  | *F1* | 97.24 | 95.03 | 95.72 | 95.53 | 85.12 | 73.75 | 73.68 | 58.26 |
|  | *Precision* | 97.53 | 95.31 | 96.17 | 95.84 | 87.7 | 77.19 | 76.85 | 59.93 |
|  | *Recall* | 97.15 | 95.04 | 95.67 | 95.54 | 84.03 | 72.68 | 72.79 | 57.92 |
| 10 | *Accuracy* | 97.8 | 93.44 | 94.24 | 95.11 | 87.54 | 82.82 | 90.5 | 73.32 |
|  | *F1* | 97.81 | 93.43 | 94.48 | 95.06 | 88.07 | 83.46 | 90.62 | 73.27 |
|  | *Precision* | 97.85 | 93.95 | 95.59 | 95.41 | 89.52 | 87 | 91.16 | 74.2 |
|  | *Recall* | 97.8 | 93.44 | 94.24 | 95.11 | 87.54 | 82.82 | 90.51 | 73.32 |
| 11 | *Accuracy* | 97.08 | 91.91 | 93.52 | 94.81 | 81.5 | 82.8 | 93.33 | 79.92 |
|  | *F1* | 97.08 | 92.05 | 94.08 | 94.77 | 82.72 | 83.47 | 93.55 | 79.32 |
|  | *Precision* | 97.17 | 92.81 | 95.81 | 95.08 | 86.53 | 87.87 | 94.27 | 80.06 |
|  | *Recall* | 97.08 | 91.91 | 93.52 | 94.81 | 81.5 | 82.8 | 93.33 | 79.92 |
| 12 | *Accuracy* | 96.42 | 96.04 | 92.95 | 95.2 | 76.37 | 80.79 | 93.08 | 80.21 |
|  | *F1* | 96.42 | 96.12 | 93.63 | 95.16 | 75.5 | 80.94 | 93.4 | 79.69 |
|  | *Precision* | 96.45 | 96.28 | 95.63 | 95.5 | 82.34 | 85.89 | 94.45 | 80.49 |
|  | *Recall* | 96.42 | 96.04 | 92.95 | 95.2 | 76.37 | 80.79 | 93.08 | 80.21 |

Table J Investigating the impact of size k in the PC-mer method generating input vectors for an LR classifier

| *k-mer* | *Metrics* | *DataSets (LR)* | | | | | | | |
| --- | --- | --- | --- | --- | --- | --- | --- | --- | --- |
|  |  | *AMP* | | | | *SG* | | | |
|  |  | *Class*  (%) | *Order*  (%) | *Family*  (%) | *Genus*  (%) | *Class*  (%) | *Order*  (%) | *Family*  (%) | *Genus*  (%) |
| 3 | *Accuracy* | 81.28 | 45.48 | 36.89 | 19.29 | 59.47 | 26.50 | 263 | 6.49 |
|  | *F1* | 79.41 | 43.19 | 32.01 | 17.42 | 57.37 | 22.10 | 13.06 | 5.10 |
|  | *Precision* | 79.60 | 44.18 | 31.58 | 17.21 | 57.46 | 22.66 | 13.97 | 5.29 |
|  | *Recall* | 81.28 | 45.48 | 36.89 | 19.29 | 59.47 | 26.50 | 263 | 6.49 |
| 4 | *Accuracy* | 86.45 | 62.18 | 54.41 | 409 | 63.61 | 33.63 | 26.47 | 180 |
|  | *F1* | 85.89 | 61.53 | 52.51 | 39.13 | 61.58 | 339 | 21.11 | 9.48 |
|  | *Precision* | 85.85 | 62.04 | 52.25 | 39.21 | 62.70 | 375 | 21.47 | 9.33 |
|  | *Recall* | 86.45 | 62.18 | 54.41 | 409 | 63.61 | 33.63 | 26.47 | 180 |
| 5 | *Accuracy* | 91.80 | 826 | 74.09 | 64.37 | 726 | 46.30 | 39.30 | 21.12 |
|  | *F1* | 91.69 | 805 | 73.78 | 64.08 | 69.29 | 44.40 | 36.20 | 213 |
|  | *Precision* | 91.66 | 824 | 73.86 | 64.43 | 69.47 | 44.48 | 36.12 | 19.99 |
|  | *Recall* | 91.80 | 826 | 74.09 | 64.37 | 726 | 46.30 | 39.30 | 21.12 |
| 6 | *Accuracy* | 96.95 | 92.10 | 89.15 | 82.63 | 83.17 | 63.66 | 58.46 | 39.14 |
|  | *F1* | 96.94 | 92.08 | 89.11 | 82.57 | 82.90 | 62.81 | 57.40 | 38.52 |
|  | *Precision* | 96.95 | 92.14 | 89.26 | 82.95 | 82.88 | 62.92 | 57.29 | 38.64 |
|  | *Recall* | 96.95 | 92.10 | 89.15 | 82.63 | 83.17 | 63.66 | 58.46 | 39.14 |
| 7 | *Accuracy* | 98.91 | 97.21 | 96.53 | 93.09 | 968 | 819 | 78.28 | 59.61 |
|  | *F1* | 98.91 | 97.21 | 96.52 | 93.08 | 963 | 79.92 | 78.11 | 59.48 |
|  | *Precision* | 98.91 | 97.23 | 96.58 | 93.26 | 961 | 79.99 | 78.29 | 608 |
|  | *Recall* | 98.91 | 97.21 | 96.53 | 93.09 | 968 | 819 | 78.28 | 59.61 |
| 8 | *Accuracy* | 99.80 | 99.45 | 99.39 | 96.95 | 96.49 | 958 | 946 | 76.59 |
|  | *F1* | 99.80 | 99.45 | 99.39 | 96.96 | 96.49 | 952 | 944 | 76.64 |
|  | *Precision* | 99.80 | 99.45 | 99.40 | 97.04 | 96.49 | 956 | 960 | 77.24 |
|  | *Recall* | 99.80 | 99.45 | 99.39 | 96.95 | 96.49 | 958 | 946 | 76.59 |
| 9 | *Accuracy* | 99.95 | 99.89 | 99.94 | 98.29 | 98.66 | 95.53 | 96.86 | 88.39 |
|  | *F1* | 99.95 | 99.89 | 99.94 | 98.29 | 98.66 | 95.52 | 96.85 | 88.39 |
|  | *Precision* | 99.95 | 99.89 | 99.94 | 98.31 | 98.66 | 95.54 | 96.90 | 88.67 |
|  | *Recall* | 99.95 | 99.89 | 99.94 | 98.29 | 98.67 | 95.53 | 96.86 | 88.39 |
| 10 | *Accuracy* | 99.99 | 99.98 | 99.99 | 98.52 | 99.69 | 98.69 | 99.31 | 94.22 |
|  | *F1* | 99.99 | 99.97 | 100 | 98.52 | 99.69 | 98.68 | 99.31 | 94.20 |
|  | *Precision* | 99.99 | 99.98 | 100 | 98.54 | 99.69 | 98.69 | 99.32 | 94.35 |
|  | *Recall* | 99.99 | 99.98 | 100 | 98.52 | 99.69 | 98.69 | 99.31 | 94.22 |
| 11 | *Accuracy* | 99.99 | 99.99 | 100 | 98.61 | 99.94 | 99.42 | 99.72 | 96.61 |
|  | *F1* | 99.99 | 99.99 | 100 | 98.60 | 99.94 | 99.41 | 99.72 | 96.58 |
|  | *Precision* | 99.99 | 99.99 | 100 | 98.62 | 99.94 | 99.41 | 99.73 | 96.69 |
|  | *Recall* | 99.99 | 99.99 | 100 | 98.61 | 99.94 | 99.42 | 99.72 | 96.61 |
| 12 | *Accuracy* | 100 | 100 | 100 | 98.64 | 100 | 99.74 | 99.87 | 97.51 |
|  | *F1* | 100 | 100 | 100 | 98.63 | 100 | 99.74 | 99.86 | 97.49 |
|  | *Precision* | 100 | 100 | 100 | 98.66 | 100 | 99.74 | 99.87 | 97.57 |
|  | *Recall* | 100 | 100 | 100 | 98.64 | 100 | 99.74 | 99.87 | 97.51 |

# Evaluating classification at the genus level utilizing LR classifier and PC-mer method

Table K Evaluating AMP and SG datasets classification at the genus level utilizing LR classifier and PC-mer method

| Datasets | Algorithm | k | Accuracy (%) | Precision (%) | Recall (%) | F1-score (%) |
| --- | --- | --- | --- | --- | --- | --- |
| AMP | CNN | 3 | 51.01 | 51.40 | 50.90 | 50.84 |
|  |  | 4 | 77.69 | 77.91 | 77.69 | 77.57 |
|  |  | 5 | 88.38 | 88.07 | 88.07 | 88.98 |
|  |  | 6 | 90.92 | 91.14 | 90.91 | 90.82 |
|  |  | 7 | 91.33 | 91.57 | 91.32 | 91.18 |
|  | DBN | 3 | 56.69 | 57.88 | 56.62 | 55.56 |
|  |  | 4 | 85.10 | 85.47 | 85.08 | 84.53 |
|  |  | 5 | 89.82 | 90.12 | 89.82 | 89.63 |
|  |  | 6 | 90.55 | 90.37 | 90.53 | 90.45 |
|  |  | 7 | 91.37 | 91.62 | 91.37 | 91.26 |
|  | RDP | - | 83.84 | 84.42 | 83.57 | 83.65 |
|  | Our Method | 3 | 19.28 | 17.21 | 19.28 | 17.42 |
|  |  | 4 | 40.09 | 39.21 | 40.09 | 39.12 |
|  |  | 5 | 64.37 | 64.42 | 64.37 | 64.08 |
|  |  | 6 | 82.62 | 82.94 | 82.62 | 82.57 |
|  |  | 7 | 93.08 | 93.26 | 93.08 | 93.08 |
|  |  | 8 | 96.95 | 97.03 | 96.95 | 96.95 |
|  |  | 9 | 98.28 | 98.31 | 98.28 | 98.28 |
|  |  | 10 | 98.52 | 98.53 | 98.52 | 98.51 |
|  |  | 11 | 98.60 | 98.61 | 98.60 | 98.60 |
|  |  | 12 | 98.63 | 98.65 | 98.63 | 98.62 |
| SG | CNN | 3 | 17.02 | 17.32 | 16.53 | 16.69 |
|  |  | 4 | 32.98 | 33.42 | 32.59 | 32.65 |
|  |  | 5 | 59.80 | 60.34 | 59.41 | 59.31 |
|  |  | 6 | 80.77 | 81.10 | 80.41 | 80.33 |
|  |  | 7 | 85.50 | 85.70 | 85.20 | 85.11 |
|  | DBN | 3 | 17.75 | 19.80 | 17.50 | 16.32 |
|  |  | 4 | 54.11 | 55.62 | 53.67 | 53.17 |
|  |  | 5 | 71.44 | 72.45 | 71.07 | 70.99 |
|  |  | 6 | 77.85 | 78.36 | 77.53 | 77.47 |
|  |  | 7 | 81.27 | 81.87 | 80.92 | 80.94 |
|  | RDP | - | 80.38 | 80.83 | 80.18 | 80.09 |
|  | Our Method | 3 | 06.49 | 05.28 | 06.49 | 05.09 |
|  |  | 4 | 10.80 | 09.33 | 10.80 | 09.47 |
|  |  | 5 | 21.12 | 19.99 | 21.12 | 20.12 |
|  |  | 6 | 39.14 | 38.64 | 39.14 | 38.51 |
|  |  | 7 | 59.61 | 60.08 | 59.61 | 59.48 |
|  |  | 8 | 76.59 | 77.24 | 76.59 | 76.64 |
|  |  | 9 | 88.39 | 88.66 | 88.39 | 88.39 |
|  |  | 10 | 94.22 | 94.34 | 94.22 | 94.19 |
|  |  | 11 | 96.61 | 96.69 | 96.61 | 96.58 |
|  |  | 12 | 97.50 | 97.57 | 97.50 | 97.48 |

# Confusion matrices of utilizing LR classifier and PC-mer method

| 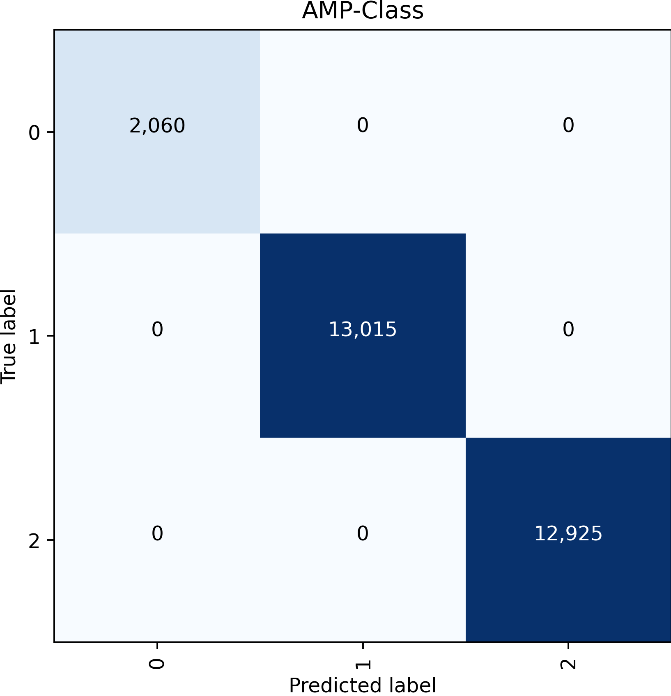  Figure E Confusion matrices of classification at the class level of AMP utilizing LR classifier and PC-mer method |
| --- |
| 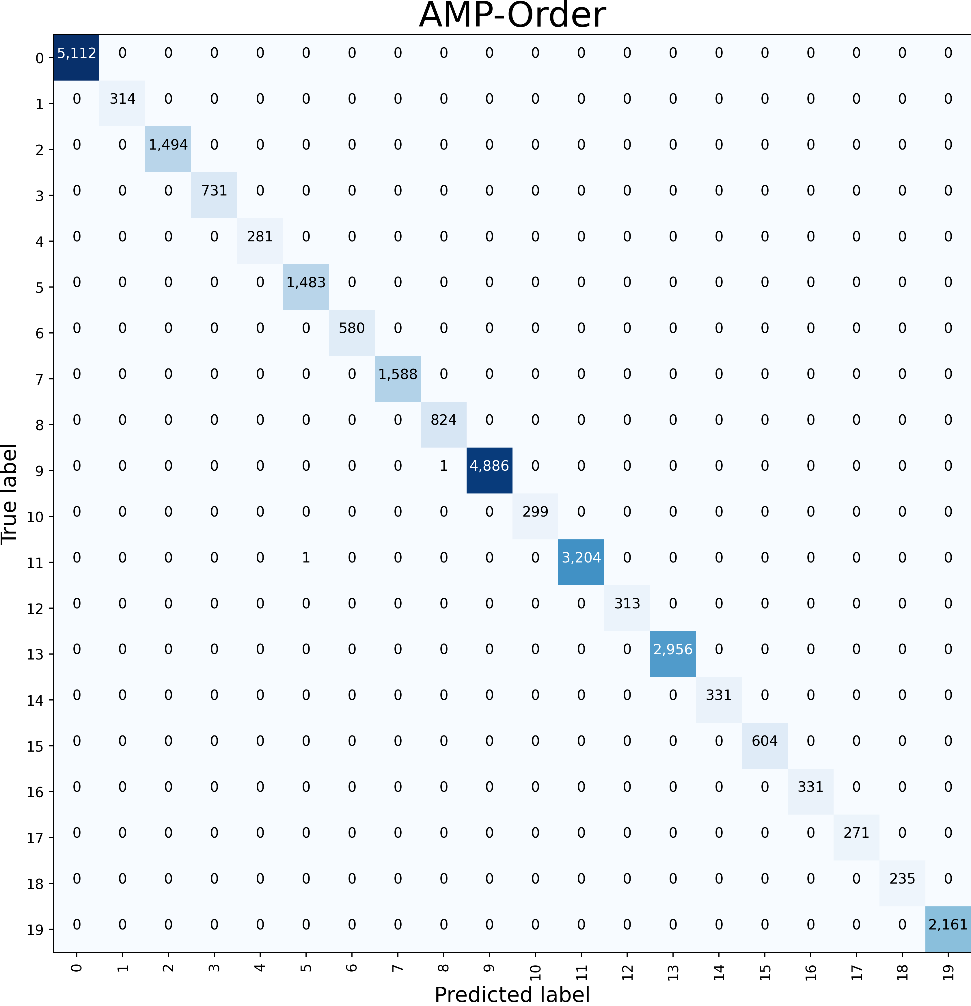  Figure F Confusion matrices of classification at the order level of AMP utilizing LR classifier and PC-mer method |


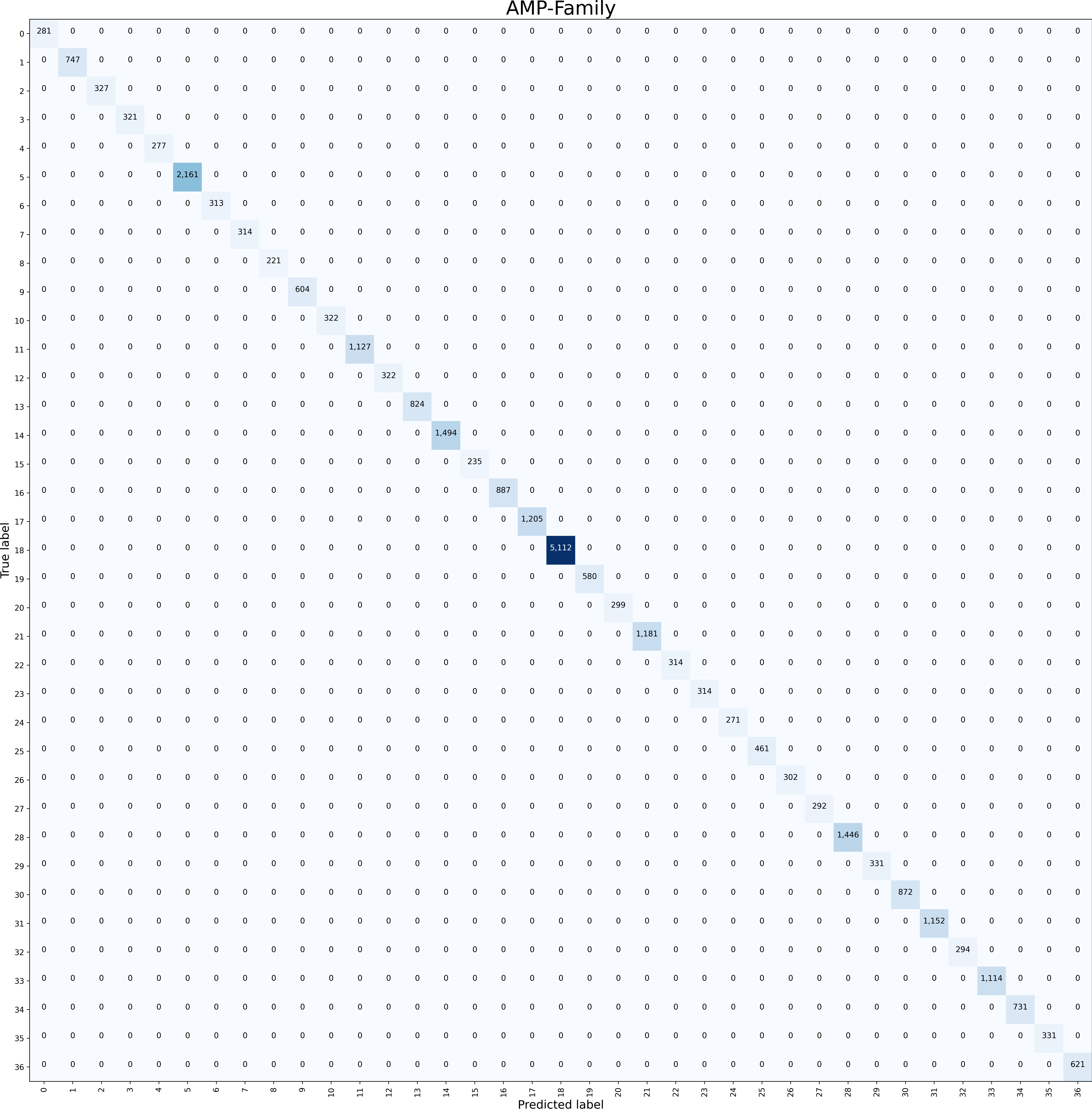


Figure G Confusion matrices of classification at the family level of AMP utilizing LR classifier and PC-mer method


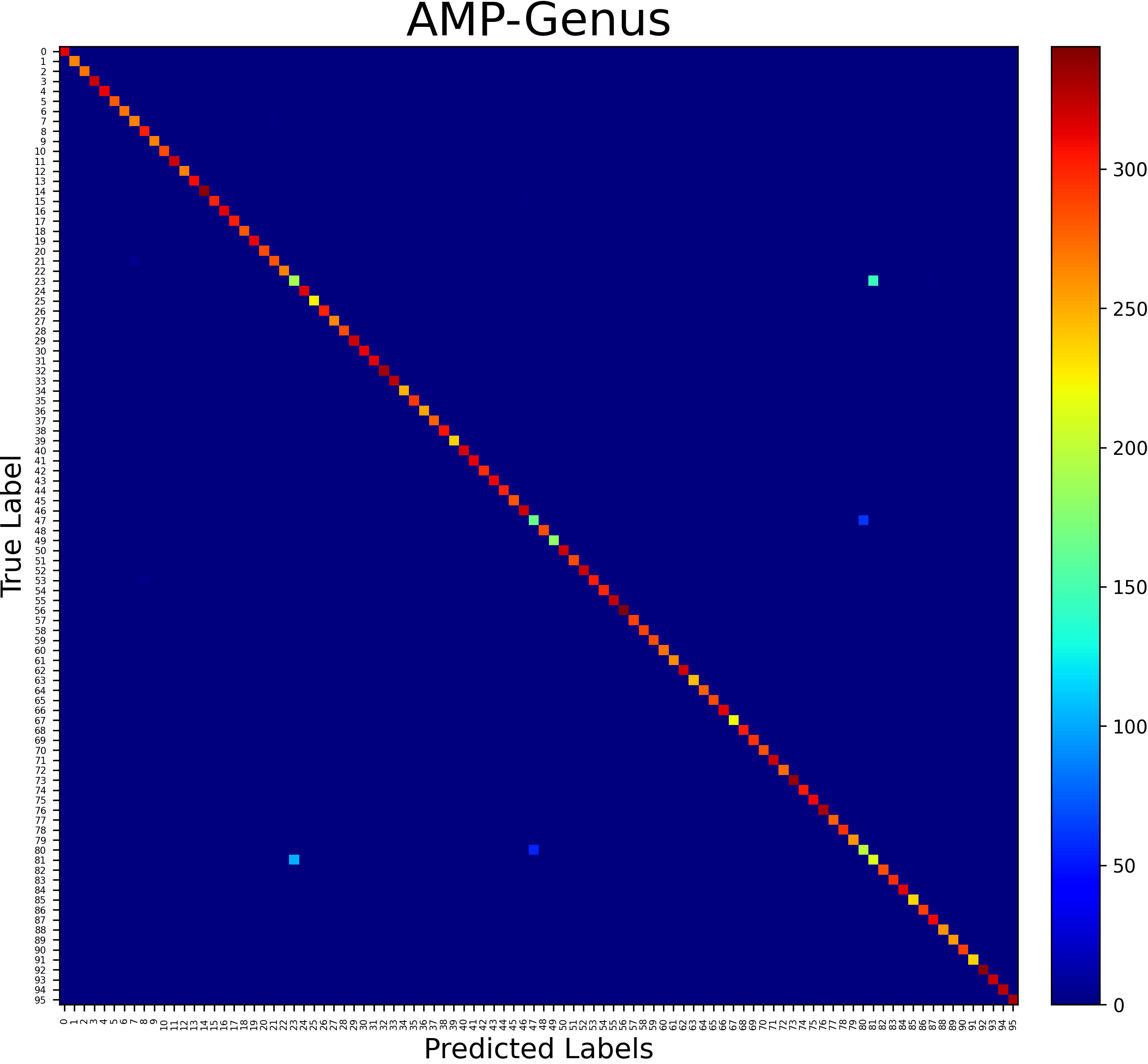


Figure HConfusion matrices of classification at the genus level of AMP utilizing LR classifier and PC-mer method

| 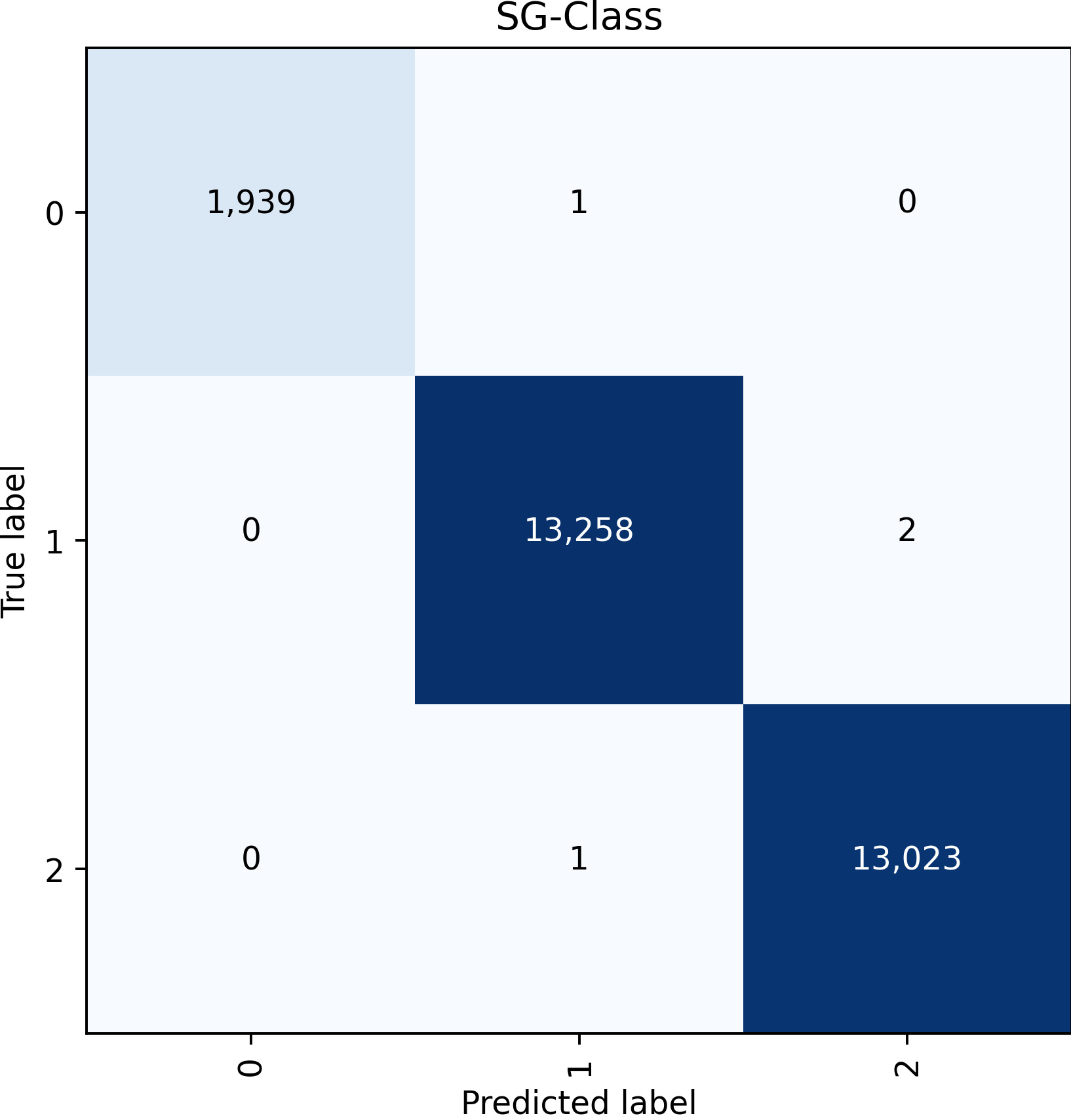 Figure I Confusion matrices of classification at the class level of SG utilizing LR classifier and PC-mer method |
| --- |
| 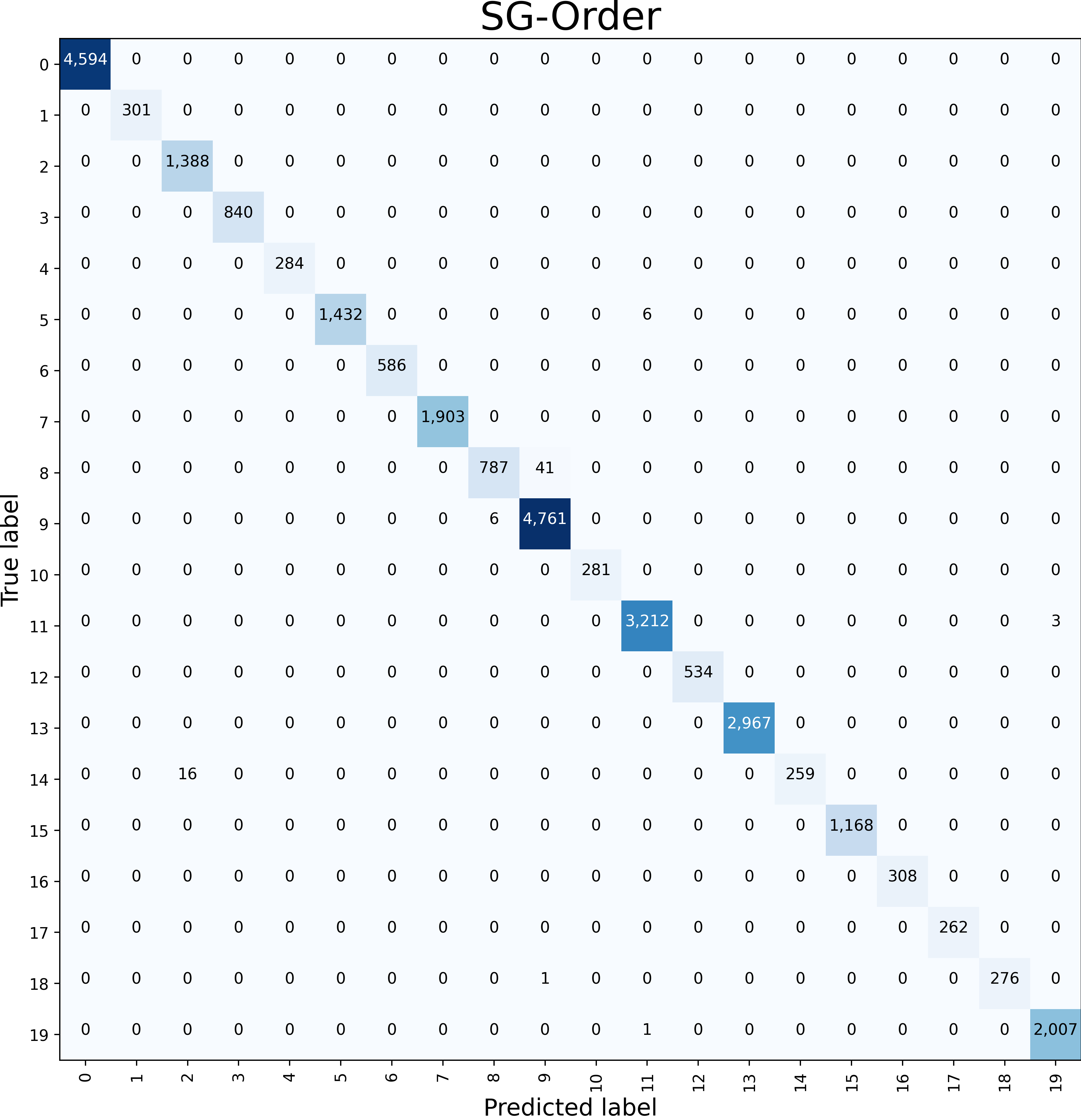 Figure J Confusion matrices of classification at the order level of SG utilizing LR classifier and PC-mer method |


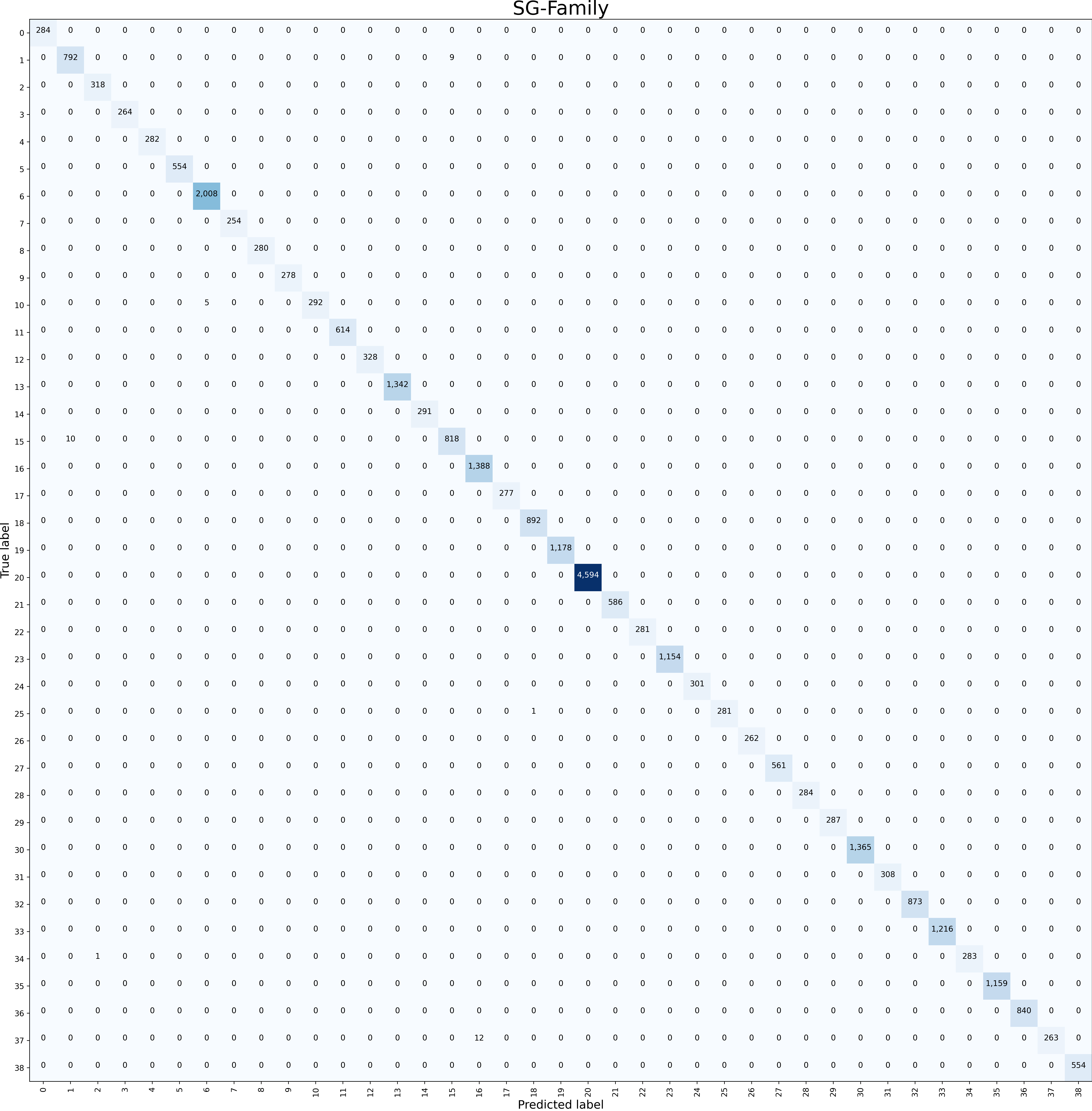


Figure K Confusion matrices of classification at the family level of SG utilizing LR classifier and PC-mer method


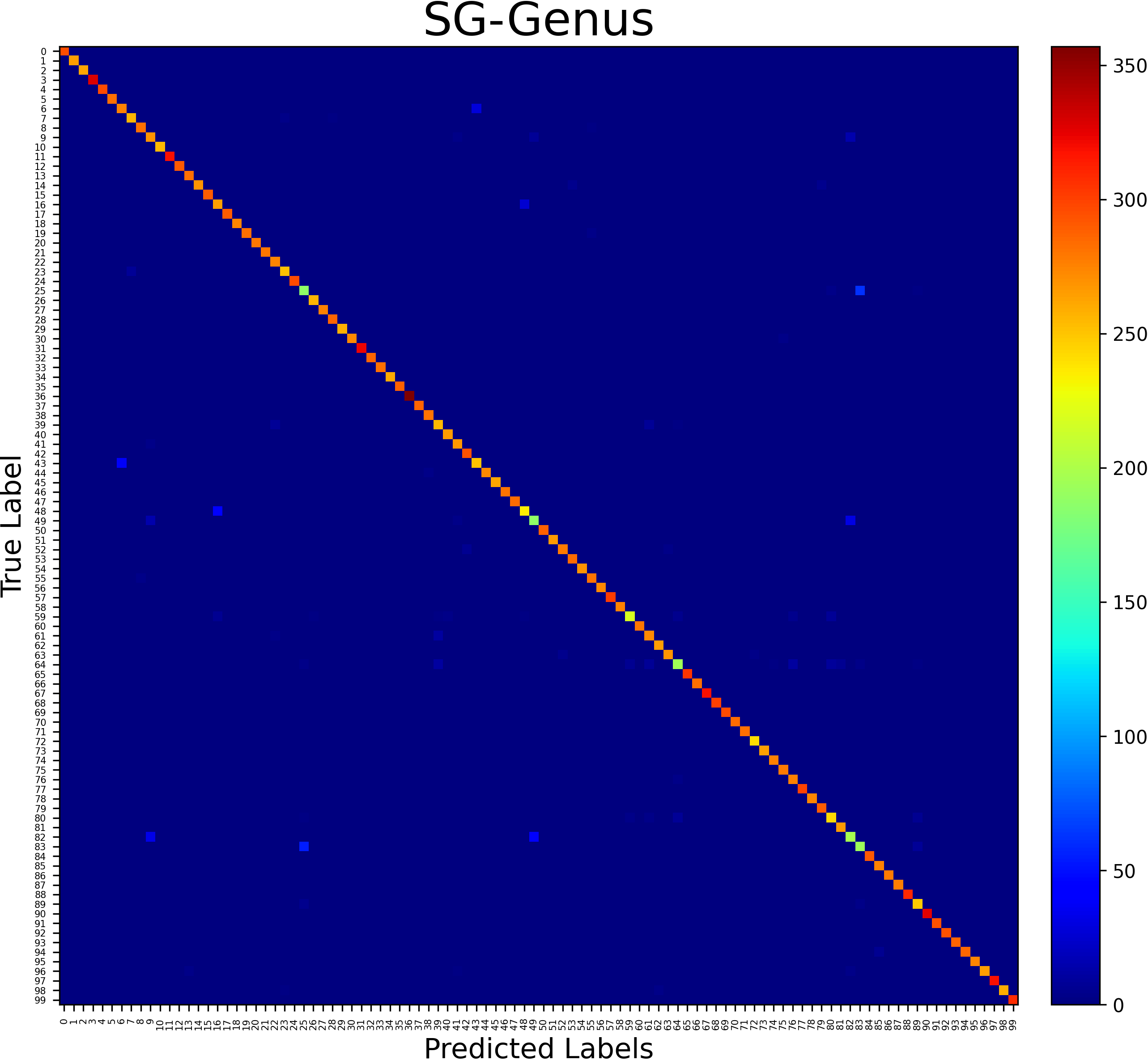


Figure L Confusion matrices of classification at the genus level of SG utilizing LR classifier and PC-mer method

# References

[1] H. J. Jeffrey, “Chaos game representation of gene structure,” *Nucleic Acids Res.*, vol. 18, pp. 2163–2170, 1990.

[2] P. J. Deschavanne, A. Giron, J. Vilain, G. Fagot, and B. Fertil, “Genomic signature: characterization and classification of species assessed by chaos game representation of sequences,” *Mol. Biol. Evol.*, vol. 16, no. 10, pp. 1391–1399, Oct. 1999.

[3] A. Fiannaca *et al.*, “Deep learning models for bacteria taxonomic classification of metagenomic data,” *BMC Bioinformatics*, vol. 19, no. S7, p. 198, Jul. 2018.

[4] C. Yuan, J. Lei, J. Cole, and Y. Sun, “Reconstructing 16S rRNA genes in metagenomic data,” *Bioinformatics*, vol. 31, no. 12, pp. i35–i43, Jun. 2015.

[5] M. Ramazzotti, L. Berná, C. Donati, and D. Cavalieri, “riboFrame: An Improved Method for Microbial Taxonomy Profiling from Non-Targeted Metagenomics,” *Front. Genet.*, vol. 6, Nov. 2015.

[6] B. D. Kaehler, N. A. Bokulich, D. McDonald, R. Knight, J. G. Caporaso, and G. A. Huttley, “Species abundance information improves sequence taxonomy classification accuracy,” *Nat. Commun.*, vol. 10, no. 1, p. 4643, Dec. 2019.
